# Applying the socioecological model to examine the beliefs, perceptions and attitudes surrounding preterm birth in Ethiopia: a qualitative study

**DOI:** 10.1136/bmjopen-2024-093030

**Published:** 2026-02-06

**Authors:** Abiy Seifu Estifanos, Meron Addis Gelaw, Hewan Getachew, Beyene Roba Ireso, Asrat Dimtse, Gesit Metaferia, Tequam Debebe Woldehawariat, Miraf Walelegn, Hema Magge, Meselech Assegid Roro, Rediet Gezahegn Gobena, Yakob Desalegn Nigatu, Yalemwork Mengistu, Bilal Shikur, Rahel Demissew, Selemawit Asfaw Beyene, Alison Tumilowicz

**Affiliations:** 1 Center for Implementation Sciences (CIS), Aklilu Lemma Institute of Health Research, Addis Ababa University, Addis Ababa, Ethiopia; 2 Department of Reproductive, Family, and Population Health, School of Public Health, Addis Ababa University, Addis Ababa, Ethiopia; 3 Department of Pediatrics and Child Health, School of Medicine, Addis Ababa University, Addis Ababa, Ethiopia; 4 Department of Pediatrics and Child Health, St Paul’s Millennium Medical College, Addis Ababa, Ethiopia; 5 Department of Radiology, School of Medicine, Addis Ababa University, Addis Ababa, Ethiopia; 6 Harvard Medical School, Boston, Massachusetts, USA; 7 Maternal, Newborn, Child Nutrition and Health, Innovation to Scale, Africa Regional Office, Gates Foundation, Seattle, Washington, USA; 8 Department of Public Health Nutrition and Dietetics, School of Public Health, Addis Ababa University, Addis Ababa, Ethiopia; 9 Department of Obstetrics and Gynecology, School of Medicine, Addis Ababa University, Addis Ababa, Ethiopia; 10 Maternal, Newborn, Child Nutrition & Health (MNCNH) Team, Gender Equality Division, Gates Foundation, Seattle, Washington, USA

**Keywords:** Ethiopia, Health policy, Community child health, QUALITATIVE RESEARCH, Delivery of Health Care, Integrated, Attitude

## Abstract

**Abstract:**

**Background:**

Premature birth is the leading cause of neonatal morbidity and mortality. Understanding perceptions, beliefs and attitudes towards preterm births, and how these factors influence care provision at health facilities and at home is crucial for improving preterm newborns’ health outcomes.

**Methods:**

We conducted an exploratory qualitative study at Batu and Meki communities in the East Shewa Zone of Oromia Region, Ethiopia. We conducted in-depth interviews (n=81) and focus group discussions (n=8) using semistructured guides. The study participants included women who had preterm births, family members, community members, healthcare workers and expert stakeholders. We audio-recorded, transcribed the interviews and coded the transcripts. We employed the socioecological model to present perceptions, beliefs and attitudes towards preterm birth at individual, interpersonal, organisational and societal levels.

**Findings:**

Giving birth to a preterm newborn is often associated with fear, stress, unhappiness, concern and worry. At the individual level, preterm newborns’ mothers often feel guilt and self-blame. Families tend to keep preterm birth a secret due to perceptions of ‘incompleteness’. At the interpersonal level, preterm newborns are often stigmatised and families are disappointed by mothers who give birth prematurely. However, some believe that preterm newborns are accepted within the community. At the organisational level, healthcare providers find the causes of preterm birth unpredictable, they do not consider preterm births prevalent, and consider some of them as abortion. There is also a common belief that preterm infants have a low survival rate, leading to the deprioritisation of their care. At the societal level, some believe preterm births are caused by divine will as punishment for sins committed by the mother, while others think they occur naturally. Preterm newborn’s death is often not acknowledged as true loss and families are discouraged from grieving.

**Conclusions:**

Our study found that the beliefs, perceptions and attitudes surrounding preterm birth, held by families, communities, healthcare providers and society at large, influence the care that preterm newborn–mother dyads receive both at home and within health facilities. Addressing these requires a multifaceted approach targeted at deeply ingrained attitudes and perceptions.

**STRENGTHS AND LIMITATIONS OF THIS STUDY**

- Diverse groups of participants representing parents, families, communities, healthcare providers, health managers, policy makers and other stakeholders were included to capture in-depth and wide-ranging perspectives.
- Socioecological model was used to systematically organise and present the findings on beliefs, perceptions and attitudes of the participants.
- While some participants appeared comfortable speaking with unfamiliar interviewers, we recognise that others’ responses may have been influenced by viewing the interviewers as outsiders.
- Although the insights generated by our study might be transferable to other settings with similar context, the study is conducted in a specific geography within the Oromia region in Ethiopia, limiting the generalisability of the findings to subnational or national contexts.
- While efforts were made to maintain neutrality in the data analysis and interpretation, authors might have introduced some level of bias in the interpretation of the data due to their incomplete understanding of the communities’ cultural and social dynamics.

## Introduction

 In Ethiopia, the neonatal mortality rate remains high. The last mini-Ethiopian Demographic and Health Survey report in 2019 estimated the neonatal mortality rate for Ethiopia at 33 deaths per 1000 live births.^1^ The Inter-agency Group for Child Mortality Estimation reported a lower figure for Ethiopia in 2022, with 27 deaths per 1000 live births, which is consistent with the estimate for the sub-Saharan region. This figure puts the country far from achieving the Sustainable Development Goal target 3.2, which aims to reduce neonatal deaths to 12 or fewer per 1000 live births by 2030.^2^

The causes of most of the neonatal deaths at global, regional, and national levels are known and preventable. Prematurity-related complications, intrapartum-related causes, and infection are the three leading causes accounting for 74% of neonatal deaths at global levels^3^ and about 90% of the neonatal deaths in Ethiopia.^4 5^ Preterm birth is the leading contributor to global mortality in children under 5.^6^ Among preterm neonates, respiratory distress syndrome (45%), neonatal infections (30%), and birth asphyxia (13%) are the top three causes of death.^7^

Of the total estimated 13.4 million annual preterm births worldwide,^8^ two-thirds occur due to spontaneous preterm birth.^9^ Spontaneous birth is a syndrome influenced by a range of factors, including social, cultural, environmental, clinical and genetic elements.^10^ Known risk factors for preterm birth encompass a range of conditions and circumstances. These include multiple pregnancies, infections, chronic health conditions, pre-eclampsia, antepartum haemorrhage, malnutrition, short birth intervals (less than 2 years), a history of preterm birth and inadequate antenatal care visits. In addition, tobacco exposure and other gynaecological and lifestyle factors contribute to the risk. These risk factors can be triggered by various mechanisms, such as inflammation, uteroplacental ischaemia or haemorrhage, uterine overdistension, stress and other immunological processes. While the specific cause for preterm birth often remains unidentified due to incomplete understanding of the underlying mechanisms, identifying women with these risks is crucial to address them through implementing targeted interventions.^911^,^13^

The risk of morbidity and mortality associated with prematurity is contingent on the gestational age of the preterm. In addition, a newborn’s chance of surviving and thriving largely depends on where they are born. A significant proportion of newborns die due to receiving no healthcare at all,^2^ and a disproportionate 80% of newborn deaths are among preterm or low birth weight newborns.^3^ Furthermore, preterm newborns who survive remain at an elevated risk of neuro-developmental impairments and educational and social difficulties in childhood and adolescence.^14^,^17^ However, the age of viability is inconsistently defined in Ethiopia’s national guidelines, leaving the frontline healthcare providers and managers without clear direction on care provision for these most vulnerable newborns. While the national Obstetric Management Protocol defined abortion as termination of births before 28 weeks of gestation,^18^ the Clinical Reference Manual for Advanced Neonatal Care recommends initiation of resuscitation for infants born without complicating comorbid conditions starting 26 weeks of gestation.^19^

In addition, although healthcare providers are presumably considered to have better perception and attitude towards preterm newborns, evidence suggests that they exhibit the same level of implicit bias as the wider population.^20^ The implicit bias, particularly towards the very premature newborns, stems from the perceived risk of prematurity-related disability and feeling of guilt for being part of allowing these babies to survive, which at times influences their care provision to these babies.^21^ In addition, implicit bias and stereotypes towards families of low socioeconomic status sometimes influence the clinical decision making of healthcare providers.^22^

On the other hand, parents’ utilisation of healthcare services and their interactions with healthcare professionals are shaped by their cultural beliefs and practices.^23^ Nonetheless, the systematic oversight of cultural influences on healthcare utilisation remains a significant barrier to achieving optimal health standards.^24^ Understanding community perceptions of preterm births is crucial for designing effective programmes aimed at improving the health outcomes of preterm newborns.^25^ While previous research in various African regions has consistently highlighted negative community attitudes towards premature newborns,^26 27^ there is a dearth of studies that comprehensively capture qualitative insights from diverse stakeholders.

This study examined perceptions, beliefs and attitudes related to preterm birth among various stakeholders within the Oromia region of Ethiopia. By shedding light on cultural nuances and community perspectives, we aimed to inform policies, strategies and programmes that enhance preterm newborn care and overall maternal and child health.

## Methods

### Study design

We employed a cross-sectional qualitative study design to explore beliefs, perceptions, and attitudes surrounding preterm birth. We chose a cross-sectional qualitative study design because it enables a deeper understanding of the beliefs, perceptions, and attitudes of health facility managers, healthcare providers, and mothers, while also allowing the generation of data within a short period of time and capturing diverse perspectives. The theory that guided our study is a phenomenological framework, which examines the individuals’ lived experiences and how they make sense of the world around them.^28^ The study was conducted as a part of formative research of implementation research to identify barriers and facilitators to scale-up and evaluate the impact of specialised lactation support on breastmilk feeding for preterm and/or low-birth weight infants.

### Study setting

This study was conducted in Batu and Meki communities in the East Shewa Zone of Oromia Region, Ethiopia, from March to September 2023. Oromia region is the largest of the twelve regional states of the Federal Democratic Republic of Ethiopia. The projected population of the region for 2023 was 40 884 000, with an estimated 1.3 million annual live births. The Batu community, which includes the Adami Tullu Jido Kombolcha district and the town of Batu, had an estimated annual birth of 9740 based on the 2020 population projection, whereas the Meki community, including Dugda district and the town of Meki, had an estimated annual birth of 7350 in 2020. Each year, an estimated 1709 preterm births occur in the two districts, accounting for 10% of all births.

### Study participants

We planned to conduct in-depth interviews (IDIs) with up to 112 participants; 52 from health facility, 48 from community, 12 representing policy makers and stakeholders and eight focus group discussions (FGDs) with women and men who recently gave birth in the Batu and Meki communities (see table 1). The final sample size for qualitative data was determined by saturation of information. The qualitative data were collected from the Batu and Meki hospitals, purposively selected two urban and two rural health centres located in the hospitals’ catchment and one urban and two rural health posts located in the health centres’ catchment. In addition, we interviewed policy makers and stakeholders at regional and national levels. The participants who could provide rich data on their experiences with preterm birth, including their beliefs, perceptions and attitudes, were purposively selected.

**Table 1:** Sample size for qualitative assessment

Data collection method	Participants	Sample size		Total
		Facility	Community	
IDIs	Mothers of preterm and/or low birth weight newborn	6	6	12
	Fathers of preterm and/or low birth weight newborn	6	6	12
	Mother-in-laws of preterm and/or low birth weight newborn		6	6
	Grandmothers of preterm and/or low birth weight newborn		6	6
	Traditional birth attendants		6	6
	Community/religious leaders		6	6
	Health development army/group (HDA)s		6	6
	Health extension workers		6	6
	Labour and delivery care providers	6		6
	Neonatal intensive care unit providers	2		2
	Antenatal care providers	6		6
	Maternal and child health unit head	6		6
	Labour and delivery unit head	6		6
	Neonatal intensive care unit head	2		2
	Regional and national policy makers	6		6
	Regional and national stakeholders	6		6
Total				112
FGDs	Women who recently gave birth in Batu and Meki communities		4	4
	Community stakeholders in Batu and Meki communities including traditional birth attendants, religious leaders, HDA members, elder men, elder women		4	4
Total				8

FGDs, Focus group discussions; IDIs, in-depth interviews.

IDIs and FGDs were sequenced to allow reflection by the next interviewees on data generated from the previous group of participants. First, mothers and family members were interviewed, followed by community members, healthcare providers and health managers. Then, FGDs were conducted with women who had recently given birth in the community and community stakeholders. Finally, policy makers and stakeholders at regional and national levels were interviewed.

Interviews were conducted at hospitals, health centres, health posts or participants’ homes. For home interviews, women and their partners who had given birth to preterm newborns at a health facility within the last 12 months were identified from hospital registers, their addresses were identified through health extension workers and community members, and they were interviewed at their homes. Women and men who had given birth to preterm newborns and were present in the Neonatal Intensive Care Units (NICUs) at the time of data collection were interviewed at the hospitals. If the mothers—and fathers-in-law of preterm newborns were present at the hospital, they were also interviewed there. When they are not present, a home visit is arranged with the newborn’s parents to conduct an interview. An interview with healthcare providers and unit heads was conducted in the hospitals or health centres. Interviews with community leaders, health extension workers, health development army (HDA) leaders and traditional birth attendants (TBAs) were conducted at health posts and in the community. FGDs with recent parents were held in the community. Interviews with regional and national policy makers and stakeholders were conducted at their respective offices.

### Data collection procedures

We conducted IDIs and FGDs face-to-face using semi-structured interview guides containing open-ended questions (online supplemental material 1). The participants were approached in person or invited via phone calls made by our team of seven research assistants, who had a minimum training in a Master of Public Health or Social Sciences and were trained in the study’s objectives and procedures for 3 days. Following the training, a pretest of the tools and data collection procedure was conducted. Each interviewer carried out interviews, transcribed them verbatim and took field notes. These were reviewed by the study investigators, leading to modifications of the tools. The interviews or discussions took place at the selected health facilities, in the community, at the participants’ homes, in the community or offices. On average, the IDIs were completed in 54 min, whereas the FGDs took 131 min. The IDIs and FGDs were carried out in Oromifa or Amharic, which are the local languages.

### Data management and analysis

Following the interviews, the digital recordings were directly translated verbatim into English. Field notes and transcripts were submitted on the same day of the interview or discussion. The study investigators provided written feedback and held frequent debriefing meetings with interviewers. This feedback was then incorporated into subsequent interviews. The transcripts and field notes were independently reviewed and coded by members of the research team using Microsoft Excel, where predefined domains derived from the research questions; “do perceptions, beliefs and attitudes towards preterm birth influence care provision at facility and home?”; guided the coding process. The coding of transcripts was an iterative process, involving ongoing discussions and regular consultations within the research team. The transcripts were further coded in-depth in Excel using a deductive process of thematic analysis.

We used the socioecological model to present the participants’ perceptions, beliefs and attitudes of towards preterm birth. The socioecological model has been previously used for qualitative studies to examine the multiple layers of factors affecting the behaviours of different groups of people.^29 30^ In the present study, individual level perceptions, beliefs and attitudes related to preterm birth emanated from the preterm newborn’s parents; the interpersonal level is mainly related to experiences of immediate family members and recent parents; the organisational level to healthcare providers and managers; and societal level to community health workers, community leaders, policy makers and stakeholders.

### Research team and reflexivity

The research team comprised 5 men and 12 women, representing a multidisciplinary group of professionals in health systems, clinical research and programme evaluation. All members brought extensive experience in qualitative research and were based in the Batu and Meki communities, Addis Ababa, and Seattle, USA. The Ethiopian team maintained strong connections with the local communities where the research was conducted.

The research team’s professional qualifications included Medical Doctors as well as master’s and PhD degrees in public health. The interviewers and FGD facilitators had substantial expertise in qualitative interviewing, were fluent in the participants’ languages, and were deeply familiar with the culture and context of the study area.

### Patient and public involvement

This study was conducted to assess stakeholders’ perceptions, beliefs and attitudes towards preterm birth at individual, interpersonal, organisational and societal levels. It did not involve patients or the public in the design, conduct, reporting, or dissemination of the study findings.

## Result

A total of 137 participants from IDIs (n=81) and 8 FGDs (n=56, 6–8 per FGD) were included in the study. Participants were predominantly female and 30-45 years old. In the IDIs, the majority of the participants were community stakeholders, while a smaller proportion were neonatal healthcare providers. The healthcare providers had experience ranging from 1 to 16 years, with 50% of having 5 or more years (see table 2).

**Table 2:** Demographic characteristic of participants

Characteristics	Frequency (%)	
	IDI participants (n=81)	FGD participants (n=56)
Gender		
Female	49 (60%)	41 (73%)
Male	30 (37%)	15 (27%)
Missing	2 (3%)	–
Age group		
15–30	21 (26%)	15 (27%)
30–45	28 (35%)	24 (43%)
45–60	13 (16%)	8 (14%)
≥60	10 (12%)	9 (16%)
Missing	9 (11%)	–
Type of participants		
Mothers who gave birth in the past 12 months	–	28 (50%)
Mothers with preterm birth in the past 12 months	12 (15%)	–
Community stakeholders	29 (36%)	28 (50%)
Maternal health workers	18 (22%)	–
Neonatal health workers	4 (5%)	–
Community health workers	18 (22%)	–

FGD, focus group discussion; IDI, in-depth interview.

This study highlights key themes, including beliefs about the causes, risk factors, and survival chances of preterm newborns, as well as community attitudes towards preterm newborns and their parents. We present these findings, categorised according to the components of the socioecological model. This model illustrates the complex interplay among individual, interpersonal, organisational, and societal aspects of beliefs, perceptions, and attitudes related to preterm birth. We also provided illustrative quotes to elucidate these themes further.

### Individual level

#### Perceived low risk of giving birth to preterm newborns

Mothers did not perceive themselves to be at risk of preterm delivery until it occurred. A few parents believed that the stress and shock associated with an elective caesarean section, or a recurrence of sexually transmitted diseases, put them at risk of preterm birth.

I went because I was sick! Then they told me I was pregnant then I went for follow-up at the health facility in our village. But I got sick before the appointment they gave me was due, 15 days ago. Then they brought me here and told me I couldn’t go home. (IDI, 38-year-old multiparous women with a preterm birth)

Around the time of her delivery, they informed her that she would be giving birth via operation, specifically on that Wednesday, due to the stress and worry caused by this news, her water leaked on Friday, leading to her giving birth. Therefore, the only possible reason for early birth is attributed to the distressing news of having to undergo an operation. (IDI, 45-year-old father of a preterm)

#### Guilt and self-blame

Parents of preterm newborns often express a preference for full-term delivery; this sentiment is particularly strong among those who have previously experienced the loss of a preterm newborn. Furthermore, mothers of preterm babies often internalise the event of premature birth, leading them to feel a sense of guilt and self-blame.

We have one baby born preterm and died. He was our first baby. He was weak when he was born like the current babies. Then he died after 6 days…as a father, I am under great stress. I feel sad. It is good if they are born by finishing their time. (IDI, father of a preterm)

I felt sad and angry. Sometimes I thought that it was my fault. I was working for a while after I knew I was pregnant to act as if I wasn’t pregnant and hide it from others. I suspected it [preterm birth] might be from that [working], and I was angry with myself. After I found out I had high blood pressure, I also thought it might be from that. Overall, I was sad because it [gestational hypertension] was additional terrifying news beside the fact that I wasn’t expecting to be pregnant. Then, even though I accepted the pregnancy and agreed to carry the baby and give birth, delivering a premature baby was also additional sad news. So, I felt sad. But now, thanks to God, he is in good health, as you can see [pointing at the baby] he is breastfeeding. (IDI, 17-year-old primiparous woman with a preterm birth)

#### Low sense of self-efficacy

Some mothers of preterm newborns have expressed the challenges they face due to premature birth. They recount their experiences of struggling to breastfeed their newborns and their concerns about the potential health and developmental issues their preterm babies might encounter, such as low birth weight or difficulties with suckling. These beliefs lead to emotional distress and a sense of insufficient self-efficacy.

I felt sad when he didn’t breastfeed. I was thinking about it the whole night because they took him to the hot room [NICU] due to his inability to breastfeed…I almost cried and felt sad because he was not breastfeeding. (IDI, woman with a preterm birth)

If they are delivered after completing the months of pregnancy, their body becomes full. But now her weight is also low. If she was delivered term, her weight would have increased. But now she was too little when she was delivered. I can do nothing but feel sad. (IDI, 32-year-old woman with a preterm birth)

#### Uncertainty in the preterm newborn survival chance and ability to grow

Parents have also expressed a lack of confidence or uncertainty about their newborns’ survival and ability to grow when they were first born. This concern is echoed by a parent who admitted, ‘I didn’t expect him to survive’. These beliefs stem from their perception of preterm newborns as ‘too little’, ‘very small’, ‘weak’, ‘not strong enough to survive’, ‘not fully developed’, ‘fragile’ and ‘incomplete’.

#### Survival of the preterm newborn is in the hands of God

Parents of preterm newborns also pointed out that the survival of their preterm newborns is ultimately in the hands of a higher power or God.

When I had a preterm birth, I felt sad because, as you know, giving birth at 8 months reduces the baby’s chances of survival. Then, I left it to God because he is the one who let them grow or takes them. (IDI, 30-year-old multiparous woman with a preterm birth)

Contrary to these beliefs, a small number of parents and family members felt more confident and less anxious about the survival of their premature babies if they had previous exposure to, or had heard or seen instances of, preterm babies who ultimately survived.

I have heard and seen this kind of case previously, [a baby] born at six months [of gestation] and that baby grew, so I didn’t feel anything new. (IDI, 32-year-old father of a preterm)

I was so happy then and they [health care providers] even told me ‘Yes they can grow’. They also told me there are even preterm newborns delivered earlier than them who grew up. I was relieved after I heard that from them. (IDI, 20-year-old sister-in-law to a mother with a preterm birth)

Similarly, a few participants highlighted the potential for preterm newborns to survive, especially if they receive appropriate healthcare and nutrition. This sentiment was conveyed by a father of a preterm who remarked, “*Preterm newborns can survive if they receive the necessary care and medications*”. Other participants also expressed similar views.

I believe preterm newborns can survive if they receive appropriate medications and sufficient care. Immediate breastfeeding and other supportive measures can increase their chances of survival. (IDI, 51-year-old grandmother of a preterm baby)

…But for the survival of preterm neonates, the facility where they are born really matters. So, if they are born in a better facility, they will have a good chance of survival. (IDI, 36-year-old senior program officer/expert stakeholder)

### Interpersonal level

#### Preterm birth is stigmatised

Many participants, particularly those from IDIs, indicated that preterm birth is not a topic that is actively discussed within the community. They further noted that preterm births are often stigmatised and do not receive significant attention. It has also been commented that some individuals even advise mothers to abandon their preterm newborns at the hospital under the assumption that it is unlikely they will survive.

They do not communicate such issues to other communities. They consider this as something shameful. They keep it secret. I don’t know the reason. I don’t know whether it is for the child’s psychology or why it is. Most of the time it is kept only in the family, and they do not communicate with other communities. I think it may be because the community says that ‘the child is born at seven months, or they are born incomplete’. (FGD, 23–41 year-old woman who gave birth in the past 12 months)

#### Family members are disappointed

A father to a preterm newborn expressed disappointment that the mother had given birth to a premature baby, leading to an extended hospital stay and indicating a general preference for a full-term birth.

If she [the mother] had some disease, I may say ‘It is because of this disease’, I could attribute it [the preterm birth] to that illness. We had to leave our home and stay here because she had given birth to preterm babies. So, we would have preferred if the baby was born at term (IDI, 38-year-old father of a preterm)

Just like the father, family members of preterm newborns also experience feelings of sadness and worry, primarily due to their fear that the newborns might not survive. A grandmother to a preterm baby also added that preterm birth is a tough ordeal for both the mother and the baby.

Well, how would you feel, when a family was eager to have a child and this kind of thing [preterm birth] happens to them. (IDI, 40-year-old religious leader)

I feel worried about premature birth. It can be a difficult and challenging experience for both the mother and the baby. These babies are often more vulnerable. It was heartbreaking to see my daughter go through this. (IDI, 68-year-old grandmother of a preterm baby

#### Focus on mothers as preterm newborns are unlikely to survive

There is a common belief among the community that preterm newborns have reduced chances of survival or will not survive at all, and parents often grapple with fear and concern about the survival of their preterm newborns, which is often associated with feelings of stress, unhappiness, fear, concern, sadness, and worry. They may feel this way due to the potential health complications and developmental issues they associate with prematurity.

Because their bodies are not as strong as those of full-term babies, these babies [premature babies] are generally weaker and more vulnerable. (IDI, 68-year-old grandmother of a preterm baby)

I definitely felt sad because I knew that premature births come with potential complications, and babies born at eight months have lower chances of survival compared to full-term babies. (IDI, 45-year-old father of a preterm)

As a result, family members often shift their focus to caring for the mother, as their hope for preterm newborns’ survival is limited.

They [the family] do nothing. If the child grows, they grow as a chance. But the major focus is given to the mother. They say, “The child may grow or may not grow, let’s save the mother.” The major focus is given to the mother. (IDI, HDA volunteer)

Furthermore, there is a tendency in the community to engage in gossip about mothers who have experienced preterm delivery, while some feel pity for them.

Yes, they will gossip about it [preterm birth], why won’t they do that? They do talk about it. They will say that, feeling sad for her unless they don’t know why that baby was born preterm. (IDI, 20-year-old sister-in-law to a mother with preterm birth)

Why do I think that way? It is because she is my person. I feel sad when such a thing [preterm birth] happens to her. (IDI, 30-year-old HDA volunteer)

#### Preterm births are increasingly accepted

On the other hand, a different perspective was presented by other participants, primarily from FGDs, arguing that preterm births are ‘accepted’ within the community and that more families of preterm newborns are coming forward in hopes of support.

Yes, they [preterm newborns] are accepted, and they help each other and support the mother of these babies. (IDI, 26-year-old neighbor of a mother with a preterm birth)

The community saw it as a curse. But now there is a change that people have started to disclose not only preterm, but they also start to disclose children with natural problems. Currently, people are starting to disclose, assuming that they may get some support. (FGD, 28–75 year-old woman who gave birth in the past 12 months)

### Organisational level

#### Preterm birth is rare and not a significant problem

Healthcare providers, particularly at the lower-level facilities, often perceive the prevalence of preterm births to be low due to the low number of deliveries in their facility. As a result, they do not consider them to be a significant concern.

We usually don’t encounter preterm births at our health center. But as of our Woreda there are cases… The total delivery is small at our health center. It’s up to 20 or 30 per month. (IDI, 32-year-old maternal health worker with 7 years’ experience)

The case of preterm is rare. I have never encountered it. Even if we get cases we refer. (IDI, 26-year-old maternal health worker 5 years’ experience)

#### Preterm birth is unpredictable

Maternal health workers alluded to the difficulty of pinpointing the cause of preterm birth by describing it as ‘unpredictable’. This unpredictability, as they explained, presents a challenge in identifying women at risk of preterm birth, thereby impacting preterm birth prevention. However, a neonatal health worker noted that within the community, prominent risk factors for preterm birth are environmental factors, malnutrition, and lack of antenatal care.

There is no exact cause for preterm. But it might be due to PROM, preeclampsia, eclampsia…it might be cervical insufficiency, or she might be RH negative. (IDI, 28-year-old neonatal health worker with 2 years’ experience)

The main cause is trauma and may be from a lack of awareness regarding ANC. They [mothers] also told me they fail to take precautions during pregnancy and lift weights beyond their strength, carry water, fall, and so on. The biggest risk factor in this area is that donkey cart rides, this ride goes [moving his arms up and down], so the amniotic fluid bursts, and the baby is born prematurely. The other thing is malnutrition is more common in mothers. (IDI, 29-year-old neonatal health worker with 5 years’ experience)

One community health worker mentioned that the use of certain medications during pregnancy would cause the mother to go into early labour.

If the mother uses what the health worker does not allow them, like medication for headaches, it may happen. It may be from God. Sometimes the mother may have intestinal parasites and if she takes medication without consulting a health professional it may happen. (IDI, 34-year-old HDA volunteer)

#### Range of perceived preterm birth risk factors and causes

However, many of the participants have also identified, with some degree of uncertainty, various risk factors associated with preterm birth, although some expressed uncertainty about these factors; some participants even considered these risk factors as potential causes of preterm pregnancy. These included pregnancy-related risk factors such as gestational hypertension, malnutrition, premature rupture of membranes, short inter-pregnancy interval, prior history of preterm birth, multiple gestations, and having a genetic predisposition to preterm birth.

I am not sure, but it may also be due to nutritional deficiency because pregnant women face many difficulties. They carry two lives, and the baby inside eats what she eats. So, if she is exposed to nutrition deficiency, preterm birth may happen. (IDI, 19-year-old father of a preterm)

I am not entirely sure, but it could be related to the discharge [leakage of discharge from her body] she experienced or her blood pressure. (IDI, 26-year-old father of a preterm)

They also identified environmental or behavioural risk factors for preterm birth, including heavy lifting, strenuous activities, physical injury, and lack of ANC during pregnancy, as well as genetic predisposition.

A woman gives birth to a preterm baby if she has a heavy workload. For example, last Friday I came across a woman who was preparing the plowed land for bean. Overnight she was bleeding, and the family took her to this health center. Her mother-in-law told me that her pregnancy is six months old. She delivered here in this health center. (FGD, 19–38 year-old woman who gave birth in the past 12 months)

If there are a lot of such premature births in her family, I think there is a chance of it happening to her. And if she has any injuries, for example, if she carries a heavy load, stumbles, or falls. (IDI, 40-year-old father of a preterm)

#### Preterm newborns have low survival chances

Similar to parents and community members, some health professionals who are at the forefront of caring for preterm newborns during birth or the neonatal period also share the belief that these newborns have a low survival rate. This opinion was echoed by a midwife who stated, “Preterm newborns have a low chance of survival.”

#### Some preterm births are considered abortions

Additionally, another midwife expressed the belief that “Babies born below a birth weight of 1.1kg are abortions.”

They also indicated that the survival of these newborns is generally given lower priority than that of the mothers. This was also confirmed by a healthcare provider from one of the hospitals.

As long as the mother is alive, no one usually questions the newborn’s survival. Especially when a preterm neonate dies, it is considered an abortion. The families don’t question, and even the hospitals are not concerned. To tell the truth, we talk about the number of neonatal deaths, but at the institutional level, I don’t think much care is given to their loss. (IDI, 43-year-old newborn and children’s health expert with 12 years’ experience)

#### Deprioritisation of care

Because preterm newborn deaths are often tolerated within the facility and the broader health system, some healthcare providers explained that care for preterm newborns is sometimes deprioritised, with greater attention directed toward saving mothers, whose deaths will be consequential and are more likely to be reported at higher levels of the system.

It is not focused on preterm labor and babies that much, but if one mother dies, it will reach the ministry of health in 24 hours, but that is not the case for preterm death (IDI, 30-year-old maternal health worker with 5 years’ experience)

Likewise, national stakeholders have confirmed the perception that preterm newborns have a lower survival rate and are prone to long-term health complications. They further explained that this perception influences healthcare practices and is widely held belief across the country.

Yes, it is common in some communities, they think low birth weight and preterm neonates have low survival rates, and will not be productive (IDI, Expert stakeholder)

### Societal level

At societal level, parents, family members, and the broader community hold perceptions, attitudes, and beliefs about preterm births, their causes, and the survival probabilities of preterm newborns. These perceptions, attitudes, and beliefs are grounded in religious, sociocultural, and health beliefs they hold about preterm birth.

#### Low survival chance and long-term adverse effects

There is widespread belief within the community, as noted by a community leader, that ‘preterm birth is shocking’ due to their perceived low survival rate and long-term adverse health issues they face. Another community leader also stated,

In our culture, [it is believed that] preterm newborns will have a deficiency. They will not have a good build or become a good baby like that of a term baby. (IDI, 45-year-old community leader)

#### Preterm newborns’ deaths are not a significant loss

Participants revealed that the community often does not perceive the death of preterm or low birth weight newborns as a significant loss. This sentiment is so pervasive that these newborns are often given notice to modest funerals in a society where funerals are typically grand events followed by extended mourning. Consequently, families are not allowed to grieve properly. This is largely because these newborns are not always considered as ‘fully human’.

Usually, the birth of low birth weight and preterm neonates is considered a negative thing, even their death is not considered as an actual death. They are not considered as full human beings. The families aren’t usually allowed to grieve; even the funeral is done in the compound. (IDI, 42-year-old newborn and child development Senior team leader/expert stakeholder)

### Punishment by God

Certain participants have expressed that preterm births are the result of the ‘will of God’ or will of the ‘rabbi’, reflecting an acceptance of preterm newborns as a divine will. On the other hand, some participants hold the belief that preterm births are simply part of the natural order, with no specific cause, “*sometimes some women give birth to preterm babies naturally*,” stated a health development army volunteer. A traditional birth attendant commented that the community, especially the older generation, believes preterm births are of spiritual consequence, a curse or punishment for sin.

Some community members attribute preterm birth to curses or other supernatural factors. They believe that certain actions or past experiences could lead to preterm birth as a form of divine punishment. However, in recent times, these beliefs may be less prevalent among the younger generation, who tend to accept things as they come without suspecting supernatural influences. (IDI, 56-year-old TBA)

### Cross-tribe marriage

Community and religious leaders have also indicated that there is a belief in the community that babies of fathers from the (Name of tribe 1) tribe are typically expected to be preterm, while those born from a father of the (Name of tribe 2) tribe are expected to be born at term. This distinction is often attributed to the perception that the (Name of tribe 2) tribe is gifted and adheres to rules such as abstaining from certain foods. Nowadays, the line between the ‘(Name of tribe 1)’ and ‘(Name of tribe 2)’ tribes is becoming blurred, thereby making the differentiation of babies born from fathers of the two tribes less clear or “confusing”.

If a woman marries into the ‘[Name of tribe 1]’ family, she’s expected to give birth at seven months. If she marries into the ‘[Name of tribe 2]’ family, she’s expected to give birth at nine months… It’s called ‘[Name of tribe 2]’ being gifted. There are rules they follow; for example, they avoid eating foods with cotton. These are cultural practices. (FGD, 50–69 years old community stakeholder)

### ‘Mich’-flu like illness and inability to fulfil pregnancy cravings

A health development army volunteer mentioned that there is a belief in the community that preterm births might be caused by pregnant women contracting “*Mich*”, an flu-like illness, during pregnancy. Another participant also noted that preterm birth can occur due to the inability of the pregnant mother to ‘fulfil pregnancy cravings’.

The women say, ‘mich’ will cause preterm delivery or abortion. [mich is caused] if you eat foods with butter and then go outside, making injera or grinding pepper out in the sun, while being pregnant. (IDI, 35-year-old multiparous woman with a preterm birth)

### Refusal to satisfy husbands’ sexual need

Another health worker from the community shared that the community gossips about the preterm mothers that they had deliberately carried heavy loads like firewood to intentionally induce preterm labour and disappoint their husbands. She added that they also believe mothers gave birth preterm because they refused to sleep with their husbands.

She carries wood so that she will have a preterm birth; she did it on purpose to disappoint her husband. But you know it could also be due to her problem. They gossip about her in secret. Even in our community, they say it [preterm birth] happened because she refused to sleep with her husband. (IDI, 30-year-old HDA volunteer)

Despite widely held belief and perception of low survival chance of preterm newborns, our data showed that the community belief was that preterm babies born at 7 months of gestation are more likely to survive and grow, while those born at 8 months have a lesser chance of survival. In addition, there is a belief that the seventh month births have a higher birth weight and are perceived as ‘normal and strong’ compared with those born on the eighth month of gestation. Participants sometimes ground these beliefs in their personal experiences. “We have never seen a baby born at eight months who has survived.”, shared a community stakeholder who participated in an FGD.

‘If you give birth at eight months, the newborn will never grow. It is better to give birth at seven months.’ They say this. (IDI, 35-year-old multiparous women with a preterm birth)

My daughter [pointing at her] was born on the seventh month, she was big. Babies born on the eighth month are small but seven month-old babies are born big…that’s what it’s believed in the community (IDI, 25-year-old multiparous woman who had preterm birth)

## Discussion

**Figure 1:**
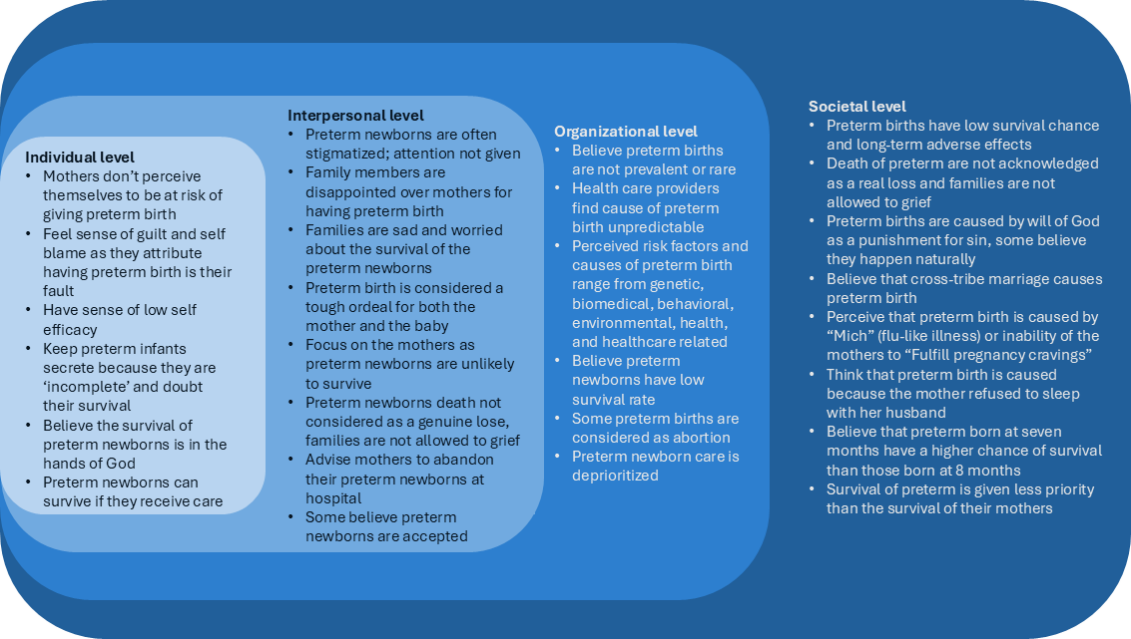
Beliefs, perceptions and attitudes towards preterm birth mapped across the socioecological model components.

In this study, we explored the perceptions, beliefs, and attitudes of parents, family members, community members, healthcare providers, health facility managers, policy makers and newborn health stakeholders regarding preterm birth. We applied the phenomenological framework to examine the lived experiences of participants and to explore how they perceive, believe, and view preterm birth and used the socioecological model to present the perceptions, beliefs and attitudes towards preterm birth at individual, interpersonal, organisational and societal levels (figure 1).

In our study, the individual-level perceptions, beliefs and attitudes held by mothers of preterm newborns towards preterm birth include feelings of guilt and self-blame for the preterm birth. Families tend to keep the preterm birth a secret due to perceptions of ‘incompleteness’ and a belief in leaving the survival chances of the newborns to divine intervention. A study conducted in England has indicated that mothers of prematurely born newborns experience more negative feelings towards their newborns when compared with mothers of full-term newborns.^31^ In addition, our study found that mothers often have a sense of low self-efficacy in caring for preterm newborns, which previous studies showed to have influenced care provision.^32^ A study from Italy further demonstrated that mothers who gave birth prior to 32 weeks of gestation experienced heightened anxiety, diminished self-perception as parents and delayed bonding with their newborns, compared with mothers who delivered after 32 weeks of gestation.^33^

Preterm newborn mothers in our study often lacked a perceived risk of having a preterm birth and readiness to provide care for the prematurely born baby. Similarly, findings from previous studies reported traumatic experiences and challenges mothers faced in provision of care for preterm newborns when they occurred.^34 35^ This could be partly due to perceived causes of preterm birth. A study conducted in Malawi assessed the perception of preterm birth among women, men, and healthcare providers. Like our study, the research identified a range of perceived causes of preterm birth, including illnesses, violence, witchcraft, ideas relating to impurity, heavy work, inadequate food, and inappropriate use of medicine. Creating a correct understanding of risk factors and causes of preterm birth among expectant parents is critical to manage their traumatic experience during birth and ready them for provision of care to the preterm newborns.

At the interpersonal level, families and communities hold certain perceptions, beliefs, and attitudes that may influence how preterm newborn–mother dyads are viewed and the care provided to them. Families are disappointed by mothers who give birth prematurely, and there is a widespread sadness about the event and concern about the preterm newborns’ survival. Given that social support is crucial for maternal resilience in provision of care for the preterm newborns,^36^ this attitude may affect the parents’ motivation and ability to provide care for their preterm newborn.

Our findings indicated that preterm birth is viewed as a significant challenge for both the mother and the baby. The focus then frequently shifts to saving the mothers due to a common belief that preterm newborns are unlikely to survive anyway, and because the health system is more sensitive to the mothers’ death. Some participants in our study even suggested that mothers should abandon their preterm newborns at the hospital. Despite some of our study participants’ report of preterm newborns’ increasing acceptance by the community, consistent with previous studies,^34 37 38^ our findings indicated that preterm births are often stigmatised and ignored. This might influence the care these vulnerable newborns receive by families and communities.

At the organisational level, our study found that healthcare providers find the causes of preterm birth to be unpredictable and they do not consider preterm births to be prevalent. The healthcare providers and managers believe that preterm newborns have a low survival rate. Although the risk of mortality increases as the gestational age of preterm newborns decreases,^39^ the general notion that preterm newborns have low survival rates might influence their care provision. A previous study from northwestern Ethiopia reported providers deprioritised care for preterm newborns because they perceived they had low survival chance.^40^ This could be driven by implicit bias in healthcare. Healthcare providers share similar implicit bias with the wider population,^20^ which influences the care provision to preterm newborns, particularly to the very preterm newborns.^21^ This bias is often rooted in their overestimation of the negative outcomes for these newborns.^21^ In addition, the decision-making processes of health professionals are sometimes influenced by non-medical factors, especially when dealing with individuals from low socioeconomic backgrounds.^22^

Furthermore, even when they are born alive, babies with a very low birth weight or gestational age are often perceived by healthcare providers as abortions, limiting their motivations to provide care. This is partly because of inconsistent definition of abortion in Ethiopia’s obstetric and newborn care guidelines.^18 19^ There is a need to make the abortion definition consistent across national maternal and neonatal healthcare guidelines in favour of allowing the premature babies as low as 26 weeks of gestation to survive and thrive as most of these babies can survive if they receive existing evidence-based interventions.^41^

At the societal level, preterm birth is often considered the mother’s fault. Some believe that preterm birth is caused by divine will as a punishment for sins committed by the mother. Some societal beliefs link cross-tribe marriages, a mother’s refusal to sleep with her husband, “Mich” or flu-like illness, and the mother’s inability to ‘fulfil pregnancy cravings’ to preterm births. Consistent with the interpersonal level, the survival of preterm newborns is often given less priority than that of their mothers. Furthermore, our finding showed that preterm newborn deaths are often not acknowledged as true losses, and families are discouraged from grieving.

Despite the differences in some areas, the unfavourable perceptions, beliefs and attitudes towards preterm birth and preterm newborns at the individual, interpersonal, organisational and societal levels are essentially similar and interconnected. We argue that these shared views, explicit and implicit biases motivate and guide the actions and inactions of those around the preterm mother-newborn dyad including husbands, family members, communities, healthcare providers, health managers, and policymakers.

In societies with patriarchal structures, such as Ethiopia, women are often unfairly blamed for negative pregnancy outcomes.^42^ This blame is deeply ingrained in the community’s beliefs and values, where women are frequently seen as the main cause of a couple’s infertility or the birth of ‘unhealthy’ newborns. Our findings indicate that families, community members, and occasionally healthcare providers attribute the cause of preterm birth to the mother’s perceived shortcomings. These can include perceived divine punishment, failure to fulfil her husband’s wishes, marrying outside of her tribe, not satisfying pregnancy cravings, acquiring ‘Mich’ or engaging in strenuous activities. As a result, they express disappointment towards the mothers and stigmatise the mother-newborn dyad. Mothers internalise these perceptions, leading to feelings of guilt and self-blame for not having a ‘complete’ newborn. Consequently, families often keep the birth of preterm newborns a secret.

We argue that these perceptions, beliefs and attitudes towards the mother-newborn dyad, particularly in cases of preterm birth, cut across individual, interpersonal, organisational, and societal levels, thereby influencing the care provided to or withheld from the preterm mother-newborn dyad. Although the health systems’ readiness to provide quality care for preterm newborns remains limited,^43^ these beliefs, perceptions, and attitudes influence home healthcare practices, the healthcare-seeking behaviour of families, the ability to use community support systems, the availability of healthcare services at facilities and the motivation and will of healthcare providers to deliver these services to preterm newborn-mother dyads. In abortion care programmes, value clarification and attitude transformation workshops have proven effective in changing the knowledge, attitudes and behavioural intentions of healthcare providers towards abortion care provision.^44 45^ In addition to awareness creation and improving the health system’s readiness for provision of care for preterm newborns, we see an opportunity for implementing similar interventions to transform the attitudes of healthcare providers and communities towards preterm birth.

Our study has strengths. The inclusion of diverse groups of study participants, including parents, families, communities, healthcare providers, health managers, policy makers and other stakeholders, has allowed us to capture in-depth and wide-ranging perspectives on preterm birth. The use of the socioecological model allowed us to systematically organise and present the findings on the beliefs, perceptions and attitudes of the study participants towards preterm birth at various levels. The study also had several limitations. First, although qualitative interviewers have deeper knowledge of the local language and culture and some of the participants appeared comfortable sharing their experiences with unfamiliar people, some of the participants’ responses might have been influenced by viewing the interviewers as outsiders. Second, although the insights our study generated might be transferable to settings with contexts similar to the study sites, the generalisability of the study findings to subnational or national contexts might be limited by the scope of the specific geography from which the data came. Finally, while the authors have made efforts to maintain neutrality in the analysis and interpretation of the data, they might have introduced some bias due to missing nuances in the local cultural and social dynamics.

### Conclusions

Our study identified that beliefs, perceptions, and attitudes surrounding preterm birth held by families, communities, healthcare providers and society at large influence the care the preterm newborn–mother dyads receive both at home and in health facilities.

To remove these implicit biases and correct unfavourable beliefs, perceptions and attitudes surrounding preterm birth among family and community members, healthcare providers, policymakers, and society at large, a multifaceted approach is required.

## Supplementary material

10.1136/bmjopen-2024-093030 online supplemental file 1

## Data Availability

Data are available on reasonable request. All data relevant to the study are included in the article or uploaded as supplementary information.
