# Supplementary material for: Applying the socioecological model to examine the beliefs, perceptions and attitudes surrounding preterm birth in Ethiopia: a qualitative study
Source: BMJ Open. 2026 Feb 6;16(2):e093030. doi: 10.1136/bmjopen-2024-093030 (PMC12887518; doi:10.1136/bmjopen-2024-093030)
Supplement: online supplemental file 1 [file bmjopen-16-2-s001.pdf]

IDI: Formative assessment-In-depth interview with mothers of Low birth weight/preterm infants

#	Item	Response options/ Codes
	Identifiers	
1	Participant ID	
2	Interview date	
3	Interviewer name	
4	Language of Interview	
5	Region	
6	Zone	
7	Woreda	
8	Health facility name	
	Background information	
9	What is your age in completed years?	
10	What is your marital status?	1. Single<br>2. Married/living together<br>3. Widowed<br>4. Separated<br>5. Other (specify)
11	How old were you when you gave birth to your first child?	
12	When did you give birth to your last child? (DD/MM/YYYY)	
13	How many live births have you had?	
14	How many of your children are alive?	
15	How many times did you give birth at health facility?	
16	How many times did you give birth at home?	
17	Where is your Place of delivery for current newborn?	
18	What is your occupation?	
19	Family size?	
20	What is the highest school grade you completed?	
21	Can you read?	1. Yes<br>2. No
22	Can you write?	1. Yes<br>2. No
23	How many times did you visit this hospital during your pregnancy, including delivery?	

Purpose: Build rapport

- To begin, I'd like to get to know you a little bit more. Tell me, what is it like for you being a mother?
    - Tell me about your family.

Purpose: understand pregnancy experience

- Tell me about your most recent pregnancy.
    - How did you find about your pregnancy?
    - Can you describe to me how you felt during your pregnancy?
    - How did you feel physically during your pregnancy?
- Did anything concern you during your pregnancy? Tell me more about this….
- Tell me about your past pregnancies… If applicable, how did this (your last) pregnancy compare to your past pregnancies?
    - How did your past pregnancy experiences affect how you felt about this pregnancy?

Purpose: understand the antenatal care experience

- Did you receive any advice during your last pregnancy?
    - What did people tell you?
- I would like to know about any care you received during your last pregnancy. Can you tell me about your experience with antenatal care starting from your first visit?
    - Describe your first antenatal care visit for me. Tell me everything that happened during this visit from the moment you arrived until you left the health center/hospital.
        - When did you first go (how far along in the pregnancy)? What made you wait that long for your first ANC?
        - Did you face any difficulties in deciding to go to antenatal care or in getting there? Tell me more…
        - Where did you go? Why did you choose to go to this facility?
        - Did anyone accompany you to antenatal care? Who? How did they help?
    - How many times during your pregnancy did you go to antenatal care?
    - What was the most interesting thing you learned at antenatal care?
    - Was there anything you wanted to know during pregnancy that you did not learn at antenatal care?
    - Did you pay for the antenatal care? How much did you pay? Do you think the payment affect utilization of antenatal care by other mothers?
- I do not know much about the health center/hospital you attended. Can you describe the space where you received the antenatal care services to me?
    - What did you like about the space?
    - What did you dislike about the space?
    - How would you describe the cleanliness?
    - What about auditory and visual privacy?
- Tell me about the staff at the health center/hospital. How did they treat you during your antenatal care follow up? Tell me about this
    - Can you give me an example?
    - How did that make you feel?
- How confident did you feel in the knowledge and skills of the health facility staff? Tell me more…
    - If applicable: how does your experience with the care compare to your prior pregnancies at a health facility?
- Was there anything particular about the antenatal care you received that you were happy with?
- Was there anything particular about the antenatal care you received that you were unhappy with?

- Overall, how satisfied would you say you were with the antenatal care that you received?
- Based on your experiences, would you use these services again? Tell me more about why or why not
- Would you recommend these services to other pregnant women? Tell me more about why or why not
- Do you have any suggestions about how the antenatal care could be improved?

Purpose: to know about gestational age dating and ACS use

- During antenatal care visit did anyone informed you about your expected date of delivery? Why?
    - How did the (midwife/nurse/doctor) know about your expected date of delivery?
- During your last pregnancy, did you have an ultrasound scan (*describe what this means*) at antenatal care? Why/why not?
    - Can you please tell me your experience with the ultrasound scan?
    - Were you informed about ultrasound scan anytime during pregnancy? What were you told? Who informed you?
    - Do you remember when you had your first ultrasound scan?
    - Tell me about your experience – what did you like and what didn't you like about the ultrasound scan?
    - What did you learn about the pregnancy after the ultrasound scan? Please describe it to me.
        - Were you informed about the gestational age of your baby after ultrasound scan?
    - Where did you receive the ultrasound scan? Which facility? Private or public?
    - Did you pay for the ultrasound scan? How much did you pay? Do you think the payment affect utilization of ultrasound by other mothers? How?
- Do you remember the last time you had your menses before you got pregnant? When?
    - Do you think pregnant women in your community remember the date for the last time they had their menses? Why/why not?
    - How can pregnant women be supported to remember the date for the last time they had their menses?
- Medication and ACS use during pregnancy
    - Were you given any medication during pregnancy or around the time of labour? What were you given? When during pregnancy was the medication given?
    - Were you informed the reasons for medications? Please tell me more.
    - Did you pay for the medication? How much did you pay? Do you think the payment affect utilization of the medication by other mothers? How?
- Do you know why you had preterm birth?
    - Did you have any previous preterm births?
    - Do you know any risk factors for preterm birth? Please tell me more.
    - Did you have any medical and pregnancy related problems during pregnancy? Any other problems related to your pregnancy?
- How did you feel when you had the preterm birth? Why did you feel that way?
    - How does your community see preterm birth? Why?
    - Are preterm infants welcomed or accepted in your community? Why?
    - In your opinion how do you see the survival of preterm infants? Why?
    - Does this affect the care you provide to your preterm infant? How?
    - What do you think should be done to improve this at the health facility and community?

Purpose: to understand about labor and childbirth and care experience

- *L&D care*: Now I would like to learn about your delivery experience. Please tell me about your delivery experience. Start from the moment you began laboring until you gave birth, if you deliver at facility, until you left the health center/hospital after giving birth.
    - Where did you give birth? How did you decide to give birth there?

    - If facility, did you face any difficulties in deciding to go to the facility for birth or in getting there?
    - How did you know you were ready to give birth?
    - Did anyone accompany you during the childbirth? Who? How did they help?
    - If you gave birth at home, who was with you? Why did you not go to a facility for birth?
    - Can you describe your experience of the care you received at birth and immediately after your baby was born?
    - Was your baby weighed after birth? Were you informed about the weight?
- I do not know much about the health center/hospital you attended. Can you describe to me the space where you received labour and delivery services?
    - What did you like about the space?
    - What did you dislike about the space?
    - How would you describe the cleanliness?
    - What about privacy?
- Tell me about the staff at the labour and delivery and postnatal care units. How did they treat you during your delivery and postnatal care? Tell me about this
    - Can you give me an example?
    - How did that make you feel?
- How confident did you feel in the knowledge and skills of the labour and delivery and postnatal care staff? Tell me more…
    - If applicable: how does your experience with the care compare to your prior deliveries at a health facility?
- Did you pay for the childbirth care, postnatal care? How much did you pay? Do you think the payment affect utilization of childbirth care by other mothers?
- Was there anything particular about the care you received (at birth and immediately after your baby was born) that you were happy with?
- Was there anything particular about the care you received (at birth and immediately after your baby was born) that you were unhappy with?
- Overall, how satisfied would you say you were with the care that you received?
- Based on your experiences, would you use the labour and delivery and postnatal care services again? Tell me more about why or why not
- Would you recommend these services to other pregnant women? Tell me more about why or why not
- Do you have any suggestions about how care at birth and immediately after birth could be improved?

Purpose: to understand the feeding experience of the low birth weight and/or preterm infant

- Can you please describe to me about the feeding experience and feeding progress of your baby starting from birth until now?
    - How was the infant fed immediately after birth? How did that make you feel?
    - When after birth was the infant fed on the breast milk?
    - If not directly fed on breast, was the baby initially given your breast milk either by tube or cup? When was tube or cup feeding initiated? If no, why not?
    - Was the baby given formula milk? If yes, why was formula milk given? When was formula milk given?
    - Was the baby given anything other than breast milk and medication (water, butter etc.)? If yes, why was he/she given?
    - Who decided on the feeding option of the baby? How?
        - Who is involved in the decision of choosing feeding options for your infants?
    - Have you expressed breast milk to feed your baby?
        - If yes, how did you feel about expressing breast milk for your infant?

        - When did you start breast milk expression (how many hours/ days after delivery)? If late initiation, why was it initiated late?
        - Do you have difficulty in breast pumping?
        - Do you feel like you have adequate milk when expressing your breast?

How many times do you express per 24 hours (day and night)?

    - How easy or difficult was it to feed your low birth weight and/or preterm infant?
    - Did you get help from anyone on feeding of your low birth weight and/or preterm infant? Please tell me more…
    - Did anyone you encourage or discourage you to feed the infant in a particular way? Tell me more…
    - Did the way your infant was fed concern you? Tell me more…
    - How do mothers of low birth weight and/or preterm infants in your community feed their infants?
- How confident did you feel in your own knowledge and skills to feed your low birth weight and/or preterm infant? Tell me more… Can you please tell me what you know about the benefits of breastfeeding?
    - Do you think breast milk or formula milk is more advantageous for low birth weight/preterm baby? Why or why not?
    - Do you think breast milk is adequate in terms of quantity and quality for your preterm infant? Why?
    - Do you think you have enough breast milk to breastfeed your baby?
    - Did you have an intention to breastfeed before your delivery?
    - Did you intend to breastfeed your baby once he/she is able to breastfeed? If no, why not?
    - Did you intend to exclusively breastfeed your baby until six months of age? If no, why not?
- How confident did you feel in the knowledge and skills of the health facility staff on supporting you with feeding of your infant? Tell me more…
    - Did you get counselling/information about feeding your baby from health care providers?
    - Were you shown how to position and attach your baby at the breast?
    - Were you shown how to express breast milk? (If she has/is expressing breast milk)
    - If applicable: how does your experience with the care compare to your prior feeding support care you received at a health facility?Is/was there any support you needed from the health care workers and the health facility? Please tell me more.
    -
- How do you compare exclusive breastmilk feeding to other feeding options (e.g., formula feeding) in terms of its effect on the survival, health, and nutritional outcomes of your infant?
- Was there anything particular about the feeding support you received that you were happy with?
- Was there anything particular about the feeding support you received that you were unhappy with?
- Overall, how satisfied would you say you were with the feeding support that you received?
- Do you have any suggestions about how the feeding of low birth weight and/or preterm infants could be improved?

Purpose: to understand the NICU care experience of the low birth weight and/or preterm infant

- Can you tell me about your experience of the care you received at the neonatal care unit? *Please probe on the below, allow the mothers to describe the care processes*
    - Skin-to-skin care
    - Medications
    - Oxygen/CPAP support
    - Referral care

    - Care for yourself
- I do not know much about the health center/hospital you attended. Can you describe to me the space where your baby received neonatal care services?
    - What did you like about the space?
    - What did you dislike about the space?
    - How would you describe the cleanliness?
    - What about privacy?
- Tell me about the staff at the neonatal care units. How did they treat you during your child's care? Tell me about this
    - Can you give me an example?
    - How did that make you feel?
- How confident did you feel in the knowledge and skills of the neonatal care staff? Tell me more…
    - If applicable: how does your experience with the care compare to your prior deliveries at a health facility?
- Did you pay for the neonatal care?
    - How much did you pay?
    - What did you pay for?
    - Do you think the payment affect utilization of neonatal care by other mothers?
- Is there anything you need to provide care for your preterm infant? (both at health facility and household level)
    - Was there anything you needed to breastfeed or express breast milk?
    - Did you face any issues regarding medication/admission of your new born?
- Was there anything particular about the care you received at neonatal care unit that you were happy with?
- Was there anything particular about the care you received at neonatal care unit that you were unhappy with?
- Overall, how satisfied would you say you were with the care that you received?
- Based on your experiences, would you use the neonatal care services again? Tell me more about why or why not
- Would you recommend these services to other mothers or families? Tell me more about why or why not
- Do you have any suggestions about how care at the neonatal care unit could be improved?

Purpose: know about the discharge process, if applicable

- How long did you stay in the facility after birth? Can you tell me about the discharge process? How was your experience about the discharge process? How was your baby at discharge from the health facility? How is s/he now?

Purpose: understand postnatal care, if applicable

- Now I would like to learn about your postnatal care experience; (if facility birth, after you were discharged from the health facility). Did you receive any follow-up visit after the delivery at the health facility or at home?
    - Please explain the visit to me from the start to finish
    - Were you informed about how to take care of your baby? Who informed you? Please tell me more about the information you received?
    - Did you face any difficulties in deciding to go to postnatal care or in getting there?
- What kind of follow-up did you expect to receive after your baby was born? Tell me more…
- What are some of the challenges that you have faced or are facing while caring for your baby? Where or from whom did you seek advice about caring for your baby?

- Did you pay for the postnatal care? How much did you pay? Do you think the payment affect utilization of newborn care by other mothers?
- Was there anything particular about the postnatal care you received that you were happy with?
- Was there anything particular about the antenatal care you that you were unhappy with?
- Overall, how satisfied would you say you were with the postnatal care that you received?
- Based on your experiences, would you use these services again? Tell me more about why or why not
- Would you recommend these services to other pregnant women? Tell me more about why or why not
- Do you have any suggestions about how the postnatal care could be improved?

IDI: Formative assessment-In-depth interview with mothers of Low birth weight/preterm infants

#	Item	Response options/ Codes
	**Identifiers**	
1	Participant ID	
2	Interview date	
3	Interviewer name	
4	Language of Interview	
5	Region	
6	Zone	
7	Woreda	
8	Health facility name	
	**Background information**	
9	What is your age in completed years?	
10	What is your marital status?	1. Single<br>2. Married/living together<br>3. Widowed<br>4. Separated<br>5. Other (specify)
11	How old were you when you gave birth to your first child?	
12	When did you give birth to your last child? (DD/MM/YYYY)	
13	How many live births have you had?	
14	How many of your children are alive?	
15	How many times did you give birth at health facility?	
16	How many times did you give birth at home?	
17	Where is your Place of delivery for current newborn?	
18	What is your occupation?	
19	Family size?	
20	What is the highest school grade you completed?	
21	Can you read?	1. Yes<br>2. No
22	Can you write?	1. Yes<br>2. No
23	How many times did you visit this hospital during your pregnancy, including delivery?	

Purpose: Build rapport

- To begin, I'd like to get to know you a little bit more. Tell me, what is it like for you being a father?
    - Tell me about your family.

Purpose: understand pregnancy experience

- Tell me about your recent pregnancy.
    - How did you find out your wife/partner was pregnant?
    - Can you describe to me how you felt when you found out about the pregnancy?
- Did anything concern you about the recent pregnancy? Tell me more about this….
- Tell me about your past pregnancies… If applicable, how did the last pregnancy compare to your previous pregnancies?
    - How did your past experience with your pregnancy experiences affect how you felt about this pregnancy?

Purpose: understand the antenatal care experience

- Did you receive any advice during the last pregnancy?
    - What did people tell you?
- How is the decision for your wife's visit of healthcare facility for antenatal care made? Who decides, why?
- Did you or any family member accompany your wife/partner for antenatal care during your last pregnancy? Why/why not?
- I would like to know about any care you received during the last pregnancy. Can you tell me about your experience with antenatal care?
    - What was the most interesting thing you learned at antenatal care? Please tell me more
    - Was there anything particular about the antenatal care services you received that you were happy with?
    - Was there anything particular about the antenatal care services you received that you were unhappy with?
    - Overall, how satisfied would you say you were with the antenatal care that you received?
    - Based on your experiences, would you use these services again? Tell me more about why or why not
    - Would you recommend these services to other couples? Tell me more about why or why not
    - Do you have any suggestions about how the antenatal care could be improved?

Purpose: to know about gestational age dating and ACS use

- Did anyone inform you about the expected date of delivery for the last pregnancy?
    - How did the (midwife/nurse/doctor) know about the expected date of delivery?
- Did your wife have an ultrasound scan (describe what this means) at antenatal care?
    - Were you informed about ultrasound scan anytime during pregnancy? What were you told? Who informed you?
    - Do you remember when your wife/partner had an ultrasound?
    - Tell me about your experience – what did you like and what didn't you like about the ultrasound scan?
    - What did you learn about the pregnancy after the ultrasound? Please describe it to me.
        - **Were you informed about the gestational age of your baby after ultrasound scan?**
    - Where did your wife/partner receive the ultrasound scan? Which facility? Private or public?
    - Did you or your wife/partner pay for the ultrasound scan? How much? Do you think the payment affect utilization of ultrasound by other mothers? How?
- Medication and ACS use during pregnancy
    - Were your wife/partner given any medication during pregnancy or around the time of labour? What was she given? When during pregnancy was the medication given?
    - Was she or you informed the reasons for medications? Please tell me more.
    - Did you pay for the medication? How much did you pay? Do you think the payment affect utilization of the medication by other mothers? How?
- Do you know why you had preterm birth?
    - Did you have any previous preterm births?
    - Do you know any risk factors for preterm birth? Please tell me more.
    - Did you have any medical and pregnancy related problems during pregnancy? Any other problems related to your pregnancy?

- How did you feel when you had the preterm birth? **Why did you feel that way?**
  - **How does your community see preterm birth? Why?**
  - **Are preterm infants welcomed or accepted in your community? Why?**
  - **In your opinion how do you see the survival of preterm infants? Why?**
  - **Does this affect the care you provide to your preterm infant? How?**
  - **What do you think should be done to improve this at the health facility and community?**

Purpose: to understand about labour and childbirth and care experience

- Now I would like to learn about your delivery experience. Tell me about your delivery experience. Start from the moment your wife/partner began laboring until she gave birth, if she delivered at facility, until she left the health center/hospital after giving birth.
  - Where did she give birth? How did you/she decide to give birth there (if at a facility)?
  - Did you face any difficulties in deciding to take your wife/partner to the facility for birth or in getting there?
  - How did you know your wife/partner was ready to give birth?
  - Did anyone accompany your wife during the childbirth? Who? How did they help?
  - If your wife/partner gave birth at home, who was with her? Why did she not go to a facility for birth?
  - Can you describe your experience of the care you received at birth and immediately after your baby was born?
  - Was your baby weighed after birth? Were you informed about the weight?

Purpose: to understand the feeding experience of the low birth weight and/or preterm infant

- Can you please describe to me about the feeding experience and feeding progress of your baby starting from birth until now?
  - How was the infant fed immediately after birth? How did that make you feel?
  - When after birth was the infant fed on the breast milk?
  - How easy or difficult was it to feed your low birth weight and/or preterm infant?
  - Did you get help from anyone on feeding of your low birth weight and/or preterm infant? Please tell me more…
  - Did anyone you encourage or discourage you to feed the infant in a particular way? Tell me more…
  - Did the way your infant was fed concern you? Tell me more…
  - How do mothers of low birth weight and/or preterm infants in your community feed their infants?
- How confident did you feel in your own knowledge and skills to feed your low birth weight and/or preterm infant? Tell me more…
- How confident did you feel in the knowledge and skills of the health facility staff on supporting you with feeding of your infant? Tell me more…
  - If applicable: how does your experience with the care compare to your prior feeding support care you received at a health facility?
  - **Is/was there any support you needed from the health care workers and the health facility**? Please tell me more.
- Tell me about the staff at the health center/hospital. How did they treat you during the delivery and your stay at the facility? Tell me about this
  - Can you give me an example?
  - How did that make you feel?
- What do you feel about your level of engagement in feeding of your low birth weight and/or preterm infant? Please tell me more…
- How do you compare exclusive breastmilk feeding to other feeding options (e.g., formula feeding) in terms of its effect on the survival, health, and nutritional outcomes of your infant?
- Was there anything particular about the feeding support you received that you were happy with?
- Was there anything particular about the feeding support you received that you were unhappy with?
- Overall, how satisfied would you say you were with the feeding support that you received?
- Do you have any suggestions about how the feeding of low birth weight and/or preterm infants could be improved?

Purpose: to understand the NICU care experience of the low birth weight and/or preterm infant

- Can you tell me about your experience of the care you received at the neonatal care unit? *Please probe on the below, allow the mothers to describe the care processes*
  - Skin-to-skin care
  - Medications
  - Oxygen/CPAP support
  - Referral care
  - Care for yourself
- Tell me about the staff at the health center/hospital. How did they treat you during your delivery and your stay at the facility? Tell me about this
  - Can you give me an example?
  - How did that make you feel?
- How confident did you feel in the knowledge and skills of the health facility staff? Tell me more…
  - If applicable: how does your experience with the care compare to your prior deliveries at a health facility?
  - Did you pay for the neonatal care? How much did you pay? **What did you pay for?**
  - Do you think the payment affect utilization of neonatal care by other mothers?
- **Is there anything you need to provide care for your preterm infant? (both at health facility and household level)**
  - **Was there anything you needed to breastfeed or express breast milk?**
  - **Did you face any issues regarding medication/admission of your new born?**
- Was there anything particular about the care you received at neonatal care unit that you were happy with?
- Was there anything particular about the care you received at neonatal care unit that you were unhappy with?
- Overall, how satisfied would you say you were with the care that you received?
- Based on your experiences, would you use the neonatal care services again? Tell me more about why or why not
- Would you recommend these services to other mothers or families? Tell me more about why or why not
- Do you have any suggestions about how care at the neonatal care unit could be improved?

Purpose: know about the discharge process, if applicable

- How long did you stay in the facility after birth? Can you tell me about the discharge process? How was your experience about the discharge process? How was your baby at discharge from the health facility? How is s/he now?

Purpose: understand postnatal care, if applicable

- Now I would like to learn about your postnatal care experience; (if facility birth, after you were discharged from the health facility). Did you receive any follow-up visit after the delivery at the health facility or at home?
  - Please explain the visit to me from the start to finish
  - Were you informed about how to take care of your baby? Who informed you? Please tell me more about the information you received?
  - Did you face any difficulties in deciding to go to postnatal care or in getting there?
- What kind of follow-up did you expect to receive after your baby was born? Tell me more…
- What are some of the challenges that you have faced or are facing while caring for your baby? Where or from whom did you seek advice about caring for your baby?
- Did you pay for the postnatal care? How much did you pay? Do you think the payment affect utilization of newborn care by other mothers?
- Was there anything particular about the postnatal care you received that you were happy with?
- Was there anything particular about the antenatal care you that you were unhappy with?
- Overall, how satisfied would you say you were with the postnatal care that you received?
- Based on your experiences, would you use these services again? Tell me more about why or why not
- Would you recommend these services to other pregnant women or families? Tell me more about why or why not
- Do you have any suggestions about how the postnatal care could be improved?

IDI: Formative assessment-In-depth interview with community members

#	Item	Response options/ Codes
	**Identifiers**	
1	Participant ID	
2	Interview date	
3	Interviewer name	
4	Language of Interview	
5	Region	
6	Zone	
7	Woreda	
8	Health facility name	
	**Background information**	
9	What is your age in completed years?	
10	What is your marital status?	1. Single<br>2. Married/living together<br>3. Widowed<br>4. Separated<br>5. Other (specify)
11	How old were you when you gave birth to your first child?	
12	When did you give birth to your last child? (DD/MM/YYYY)	
13	How many live births have you had?	
14	How many of your children are alive?	
15	How many times did you give birth at health facility?	
16	How many times did you give birth at home?	
17	Where is your Place of delivery for current newborn?	
18	What is your occupation?	
19	Family size?	
20	What is the highest school grade you completed?	
21	Can you read?	1. Yes<br>2. No
22	Can you write?	1. Yes<br>2. No
23	How many times did you visit this hospital during your pregnancy, including delivery?	

Purpose: Build rapport

- To begin, I'd like to get to know you a little bit more. Tell me, what is it like for you being [INSERT THEIR ROLE]?
    - Tell me about your family.

Purpose: understand experience of pregnant women in the community

- How do women in your community find out about their pregnancy?
- Does anything concern you about pregnant women? Tell me more about this….

Purpose: understand the antenatal care experience of pregnant women

- Do pregnant women in your community receive any advice during pregnancy? Who gives the advice? Tell me more…
- I would like to know about any care pregnant women in your community receive?
    - When do pregnant women in your community seek antenatal care? What makes pregnant women wait that long to seek antenatal care?
    - Who decides on whether a pregnant woman go to antenatal care? Who is involved in making the deciding?
    - Do pregnant women face any difficulties in deciding to go to antenatal care or in getting there? Tell me more…
    - How many times during pregnancy do women typically go to antenatal care?
- What do you think affects antenatal care use at facility by pregnant women? (Probe on the following)
    - The facility setting – space
    - Health workers' knowledge, skills, treatment of pregnant mothers
    - Payment for antenatal care service
    - Availability of transport to health facility
    - Anything else…
- Do you have any suggestions about how the antenatal care could be improved?

Purpose: to know about gestational age dating and ACS use

- How do pregnant women in your community know about their expected date of delivery? Why/why not?
- Do women in your community have access to ultrasound scan (*describe what this means*) at antenatal care?
    - Where do pregnant women receive the ultrasound scan? Which facility? Private or public?
    - How do you feel about pregnant getting ultrasound scan? Why do you feel so? Please tell me more…
    - Do they pay for the ultrasound scan? How much? Do you think the payment affect utilization of ultrasound by other mothers? How?
- Do pregnant women in your community remember the last time they had their menses before they got pregnant? Why/why not?
- Do you know any medication given to pregnant women who had preterm or low birth weight birth during pregnancy or around the time of labour? Why are the medications given for? Please tell me more…
    - Do women pay for the medication? How much? What do they pay for? Do you think the payment affect utilization of the medication by other mothers? How?
- Do you know why some women have preterm birth? Please tell me more…
- How do you feel about preterm birth? Why do you feel so? Please tell me more…
- In your community what do families do when they give birth to a preterm or low birth weight infant? Please tell me more…
    - Is the care for preterm or low birth weight infants different from the term infants? How is it different? Why is it different?
    - **How does your community see preterm birth? Why?**
    - **Are preterm infants welcomed or accepted in your community? Why?**
    - **In your opinion how do you see the survival of preterm infants? Why?**
    - **Does this affect the care provided to the preterm infant? How?**
    - **What do you think should be done to improve this at the health facility and community?**

Purpose: to understand about labour and childbirth and care experience

- Where do most pregnant women in your community give birth? Why do they choose this place?

    - Do pregnant mothers seek facility delivery? Why/why not? Please tell me more…
    - Who decides on whether a pregnant woman go to facility for childbirth care?
    - Do pregnant women face any difficulties in deciding to go to childbirth care at facility or in getting there? Tell me more…
    - Do pregnant women receive any care or support from the community when they give birth? For themselves and for their neonates? Please tell me more…
- What do you think affects care seeking by pregnant women for childbirth care at facility? (Probe on the following)
    - The facility setting – space
    - Health workers' knowledge, skills, treatment of pregnant mothers
    - Payment for childbirth care service
    - Availability of transport to health facility
    - Anything else…
- Do you have any suggestions about how the childbirth care could be improved?

Purpose: to understand the feeding experience of the low birth weight and/or preterm infant

- Can you please describe to me how preterm and/or low birth weight infants are fed in your community?
    - What do mothers or family members give to the infant immediately after birth? Why do they give? Please tell me more…
    - When after birth are they fed on the breast milk?
    - How easy or difficult was it to feed low birth weight and/or preterm infant?
    - Do mothers get help from anyone on feeding of their low birth weight and/or preterm infant? Please tell me more…
    - Does anyone encourage or discourage mothers of preterm or low birth weight infants to feed the infant in a particular way? Tell me more…
- What do you think affects breast milk feeding of preterm or low birth weight infants? (Probe on the following)
    - The facility setting – space, refrigerator, breast pump, feeding cup
    - Health workers' knowledge, skills, treatment of pregnant mothers
    - Payment for breast milk feeding support service
    - Anything else…
- How do you compare exclusive breastmilk feeding to other feeding options (e.g., formula feeding) in terms of its effect on the survival, health, and nutritional outcomes of your infant?
- Do you have any suggestions about how the feeding of low birth weight and/or preterm infants could be improved?

Purpose: understand postnatal care experience of recent mothers

- Now I would like to learn about postnatal care experience of recent mothers in your community. Do recent mothers in your community receive any follow-up visit after the delivery at the health facility or at home?
    - Who do the postnatal care visit? What do they do to the mother and the neonate during the visit?
    - Do recent mothers face any difficulties in deciding to go to postnatal care or in getting there? Please tell me more…
- What kind of follow-up did you expect recent mothers to receive after delivery? Tell me more…
- What are some of the challenges that recent mothers face while caring for their baby? Where or from whom did they seek advice about caring for their baby? Are the challenges different for recent mothers who may have preterm or low birth weight infant? How?
- Did mothers or families pay for the postnatal care? How much? Do you think the payment affect utilization of newborn care by other mothers?
- Do you have any suggestions about how the postnatal care could be improved?

FGDs with women who recently gave birth

***[Instructions to the FGD team:** Two people will be present during the FGD, comprising of one moderator and one note-taker (observer).*

Participants in this FGD are women who gave birth in the last year, including women of all ages.

Introduction

Thank you very much for taking the time to speak to us today. My name is [interviewer name], and I am one of the ACS-IR team members. Before we begin, can I please confirm that you have received a copy of the study information sheet and consent form?

As a reminder, this study aims to explore preterm birth in communities like yours. As part of this study, we are interested in hearing about women's experience during pregnancy and birth, about newborn care and about women's knowledge about preterm birth. You are free to participate in the discussion with as much or as little detail as you wish, to not respond to any questions you do not wish to answer, and to pause or leave the discussion at any time if needed.

There are no right or wrong answers. Everything you say will be treated confidentially and will not be shared outside of this group, or anyone outside of the ACS-IR study team. We would like to ask you to respect this confidentiality and also not share what others have said.

This focus group discussion will take approximately 60 to 90 minutes - depending on how much you have to say. Can I please check you are all free at the moment to talk for this amount of time?

I would also like to record our conversation- so that we can capture your responses accurately, and verify our notes. Can I confirm you are happy for me to start recording?

Thank you.

Information to collect (prior to starting and at close)

Participant sociodemographic – for each participant, list:

- **Area/community:**
- **Woman's Age:**
- **Date of baby's birth:**
- **Total number of living children**
- **Marital status:**
- **Education level:**

Information about focus group:

- **Interview start time**
- **Interview end time:**
- **Duration of interview:**
- **Interviewer name:**
- **Notetaker name:**

Antenatal care

In this section, we would like to understand women's experience in pregnancy and their care-seeking as well as their knowledge on certain pregnancy issues.

1. In your community, how do most women realise they are pregnant? What do they do to confirm they are pregnant?

2. During pregnancy, where do pregnant women receive health advice? Explain who do they go to and for what type of advice do they go to each person.

3. We would like to hear a little bit about women and their care-seeking during pregnancy:
    a. How do women decide to go to antenatal care?
    b. When do most women first seek antenatal care? How far along in pregnancy?
    c. Why do some women go to antenatal care late (e.g. 4+ months into pregnancy)?

d. How many times do women go for antenatal care before birth?
e. Why do some women go for antenatal care?
f. Why do some women not go at all for antenatal care? Why is that?
g. How do husbands/partners and the family support women to make sure they get their antenatal care visits?
h. What factors influence whether women go for antenatal care? Probe:
    i. The setting/place where antenatal care is provided
    ii. Availability of transportation
    iii. Distance to the place where antenatal care is provided
    iv. Weather conditions
    v. Long wait times
    vi. Health workers' knowledge, skills,
    vii. Treatment of women by health workers
    viii. Cost/payment for services
    ix. Family or other home responsibilities
    x. Lack of husband/family support
    xi. Anything else

4. How do pregnant women know how many months pregnant they are/ the approximate month of when their baby is due to be born?

5. We would like to discuss a woman having an ultrasound during antenatal care. Have you heard about this? Do you know why they would have an ultrasound done?
    a. Why do women seek ultrasound scanning during their pregnancy?
    b. Why do women do not seek ultrasound scanning during their pregnancy?
    c. What are some of the reasons why women get an ultrasound (probe: about gestational age dating / to know how far along their pregnancy is?)
    d. How important do women think it is to get an ultrasound?
    e. How do others in the community feel about ultrasound? Do families support women to get one?
    f. In what month of pregnancy do women usually have a first ultrasound (how many weeks or months into their pregnancy or in which antenatal care visit)? Why do they go at that time?
        i. Probe: do women prefer to wait are women until later in the pregnancy before seeking an ultrasound– if so why?
    g. Where do women go to get the ultrasound? Why do they prefer/go to this facility for the ultrasound?
    h. How many ultrasound examinations on average do women get during their pregnancy?
    i. Who tells women about ultrasounds, where do women hear about ultrasound?
    j. Who/what encourages them to have an ultrasound?
    k. Do women have to pay for an ultrasound? If so how much?
    l. Is it easy for women to get an ultrasound? If yes, why? If no, why not?
    m. Are there some women who do not get an ultrasound? Who are women who do not usually get one? Why is that? [**Note**: Probe for different challenges including availability of ultrasound services, opening hours, location, cost, fear, other reasons.]

6. Are there supports that have been done for women in your community to receive antenatal care early in pregnancy (during the first three months)? If the health services wanted to support women in your community to receive antenatal care early in pregnancy (during the first three months), what do you suggest be done to support women going?

7. Are there supports that have been done for women in the community to get an early ultrasound (in the first 24 weeks of pregnancy)? If the health services wanted to support women in your community to get an early ultrasound (in the first 24 weeks of pregnancy) because this would assess how many weeks of pregnancy/how old the baby is, what do you suggest be done to support this?

Preterm birth

In this next part of the discussion, we are trying to understand perspectives about when women go into labour early and the baby may be born too soon/before the due date. We want to understand how women in your community know this may be happening and why people think this happens. ***Note to facilitator****: If there is a specific terminology in your community used for preterm birth, please use this*

or ask the group how they call it.

8. In your opinion, when is a baby born too soon? At what age/how many weeks or months during pregnancy is a baby born too soon?
    a. Do people expect most babies to be born after nine months of pregnancy? How do you call this in this community?

9. What do people in your community think about babies who are born too soon/ or before their due date (e.g. before nine months)?
    a. What are some of the common characteristics of a baby that is born too soon? (**Note**: Probe for small, low birth weight, has complications, delicate, unable to breastfeed, etc.)
    b. How do people in the community discuss and react towards a woman who has a preterm labour and gives birth to a preterm baby? Would most people find out about it or does a family not share this? If the family do not share the preterm birth, why is the reason?
    c. How do people in the community treat a woman and her family if there is a preterm birth? Do people in your community worry about babies that are born too soon (preterm birth)? Why or why not?
    d. What do people in your community think are some of the reasons for having a preterm baby? What do they think causes a baby to be born before its due date?
    e. What do the community do to a woman with a preterm labor? Would women in this case go to a health facility? Would they go to a different facility from where they would go for birth if it was not too soon?
    f. What do people in your community think are some of the ways to prevent preterm birth or early onset of labour?
    g. Who do women who have a baby born too soon receive support from? Can they count on their husband, their family, their neighbours for support? Where do they go to get health advice?

10. Have you heard of any medications that are given to women who have gone into early labour / before the time the baby is due? If so, what medications?
    a. When and why are these medications given?
    b. Do you think women in your community would take any medication to stop giving birth too soon? Why or why not? What might influence their decision?
    c. The baby born too early may not be able to breathe on their own, as their lungs may not have been fully developed. Have you heard of any medicines to help a baby breathe at birth (e.g. steroids)? What can you tell me about these medicines?
        i. Are these medicines available for women in your community? Where and how do women who go into labour before their due date get them?
        ii. Are there women who may not get them? Why not?
    d. What do you think might influence whether a woman with a high chance of giving birth to a preterm infant to take a medication to help the preterm infant breathe better after birth?

Childbirth care

In this section, we would like to understand where women go for childbirth care and how they feel about the care they receive; we also want to know about women who give birth to a preterm baby (born too soon) or born small.

11. Where do most women go for childbirth care?
    - In the health facility? Which facility do most pregnant women go to for childbirth care? Why do they choose this facility?
    - Is it easy for women to get to the facility? [**Note**: Probe for any challenges in going to the facility care including transport, distance, cost, lack of family support.]
    - How do women feel about the care they receive in this facility?
    - Are husbands and families invited to participate in the care of the newborn while the woman and baby are in the facility? What do they do and do not? Why/why not? d
    - How long after birth do a woman and her baby get discharged?
    - Do most women feel they are oriented on how to take care of their baby before being discharged to the home? What is the orientation given to them on discharge? Do they feel confident to go home with their baby? Why or why not?
    - If women, do not go to a health facility, who supports them with home birth?

- Do women in the community who delivered in the health facility receive appointment for follow-up visit? Do they go on the appointment date?
- Do women in the community receive any follow-up visit after the delivery at home?

12. Where do most women go for childbirth care if they are having a preterm birth?
    - To a health facility? To which facility would most pregnant women go if they went into labour before their due date? How to they go to this facility? Why do they choose this facility?
    - Is it easy for women to get to this facility? [**Note**: Probe for any challenges in going to the facility care including transport, distance, cost, lack of family support.]
    - How do women feel about the care they receive in this facility?
    - Are husbands and families invited to participate in the care of the newborn while the woman and baby are in the facility?
    - How long after birth do a woman and her baby that is born too soon get discharged?
    - Do most women who have a preterm birth feel they are oriented on how to take care of their baby before discharge to the home? What is the orientation given to them on discharge? Do they feel confident to go home with their baby? Why or why not?

13. If a woman has a home birth and has a baby that is born too soon, can you tell us what happens? [**Note**: Probe – does she get referred to a health facility? Does she usually get to the facility? If not who takes care of the woman and the baby?]

Newborn care

In this section, we would like to understand the care of newborns, including newborns who are born too soon.

14. Once a woman and baby are back in the home, who helps the woman to take care of the baby?

- probe; Washing, feeding,
- What are the challenges in taking care of the baby, what do they do to overcome the challenges?

15. Who do most women go to get health advice on caring for their baby?

16. If a woman in your community gives birth to a baby born too soon, what are some of the challenges she has? Who helps her to care for the preterm baby? How do they help? In your opinion, do women need additional support to care for preterm babies

17. Who do you think we could involve from the community to help in improving how preterm babies are taken care of and to better support mothers to care for their babies? [probe; community stakeholder, health extension worker, health development army]
    a. How could we engage different community stakeholders and which should we engage?

Wrapping up

18. Are there any other issues we have not discussed that you feel are important and that we should discuss?

Closing

Thank you for your time today. Your contributions will support the health services in better understanding how to improve maternal and newborn health, including the health of women who have preterm labour and their babies.

FGDs with community stakeholders

***[Instructions to the FGD team:** Two people will be present during this FGD, comprising of one FGD moderator and one note-taker (observer).*

Participants in this FGD are people who are considered leaders in the community, including elders, traditional birth attendants, influential people in the villages, and local authorities. Note, depending on the context, TBAs may be included in the focus group or IDIs with community health workers.

Thank you very much for taking the time to speak to me today. My name is [FGD moderator name], and I am one of the ACS-IR research team members. Before we begin, can I please confirm that you have received a copy of the study information sheet and consent form?

As a reminder, this study aims to explore women's experiences and care-seeking behaviors on preterm birth in communities like yours. We are interested in hearing your views and experiences about what currently happens, and what factors influence how the onset of preterm labour is detected and managed. You are free to participate in the discussion with in as much or as little detail as you wish, to not respond to any questions you do not wish to answer, and to pause or leave the discussion at any time if needed.

There are no right or wrong answers. Everything you say will be treated confidentially and will not be shared with any of your colleagues, or anyone outside of the ACS-IR study team. We would like to ask you to respect this confidentiality and also not share what others have said.

This focus group discussion will take approximately 60 to 90 minutes - depending on how much you have to say. Can I please check you are free at the moment to talk for this amount of time?

We would also like to record our conversation- so that we can capture the conversation accurately, and compare with our notes. Can I confirm you are happy for us to start recording?

Thank you.

Participant sociodemographic – for each participant list:

- **Age/age range:**
- **Area/community:**
- **Type of community actor:**

Information about focus group:

- **Interview start time:**
- **Interview end time:**
- **Duration of interview:**
- **Interviewer name:**
- **Notetaker name**

Antenatal care

In this first discussion, we would like to understand when women in your community first seek antenatal care during pregnancy, why some women don't seek antenatal care or seek antenatal care late, and how women know how far along they are in their pregnancy.

1. In your community, how do women realise they are pregnant? What do they do to confirm they are pregnant?

2. During pregnancy, where do pregnant women receive health care advice? Explain who do they go to and for what type of advice do they go to each person for.

3. We would like to hear a little bit about when women and their care-seeking during pregnancy?
    a. How do women decide to go to antenatal care?
    b. When do most women first seek antenatal care? How far along in pregnancy?
    c. Why do some women go to antenatal care late (e.g. 4+ months into pregnancy)?
    d. How many times do they usually go to for antenatal care before birth?
    e. Why do some women go for antenatal care?
    f. Why do some women not go to at all for antenatal care? Why is that?
    g. How do husbands/partners and the family support women to make sure they get their antenatal care visits?
    h. What factors influence whether women go to antenatal care? Probe:
        i. The setting/place where antenatal care is provided
        ii. Availability of transportation
        iii. Distance to the place where antenatal care is provided
        iv. Health worker knowledge, skills,
        v. Treatment of women by health workers
        vi. Cost/payment for services
        vii. Family or home responsibilities
        viii. Lack of family support
        ix. Anything else

Instructions to the interviewer: *if the questions seem to be difficult to be answered by men, focus on probing the following questions:*

a. Do women go to antenatal care in your community? Why?
b. When do they normally go for their first antenatal care? How far along in pregnancy?
c. What are the reasons for women not attending antenatal care in your community?

19. When women are pregnant in your community, how do they know how far along they are in their pregnancy? How do pregnant women know how many months pregnant they are?

20. We would like to discuss a woman having an ultrasound during antenatal care. Have you heard about this? Do you know why they would have an ultrasound done?
    a. Why do women seek ultrasound scanning during their pregnancy?
    b. Why do women do not seek ultrasound scanning during their pregnancy?
    c. What are some of the reasons why women get an ultrasound (probe: about gestational age dating / to know how far along their pregnancy is?)
    d. How important do women think it is to get an ultrasound?
    e. How do others feel about ultrasound? Does the family support her to get one?
    f. In what month of pregnancy do women usually have a first ultrasound (how many weeks or months into their pregnancy or in which antenatal care visit)?
        a. Probe: are women waiting for pregnancy to be established before seeking care – if so why?
    g. Where do women get the ultrasound? Why do they prefer/go to this facility for the ultrasound?
        a. Why do women go to this place? How far is this facility?
        b. Does she go alone? Who takes her to this facility?
        c. Is it easy or difficult for a woman to get an ultrasound? Why?
    h. How many ultrasound examinations on average do they get during pregnancy??
    i. Who advises women or encourages them to go for the ultrasound?
    j. Do women have to pay for an ultrasound? If so, how much?

k. Are there some women who do not get an ultrasound? Who are women who do not usually get one? Why is that? [Note: Probe for different challenges including availability of ultrasound services, opening hours, location, cost, fear, other reasons.]

7. Are there supports that have been done for women in your community to receive antenatal care early in pregnancy (during the first three months)? If the health services wanted to support women in your community to receive antenatal care early in pregnancy (during the first 3 months), what do you suggest be done to support women going?

8. Are there supports that have been done for women in the community to get an early ultrasound (in the first 24 weeks of pregnancy)? If the health services wanted to support women in your community to get an early ultrasound (in the first 24 weeks / 6 months of pregnancy) because this would assess how old the baby is, what do you suggest be done to support this?

Preterm birth and danger signs

In this next part of the discussion, we are trying to understand perspectives about when women go into labour early and the baby may be born too soon. We want to understand how women in your community know this may be happening and why it happens. If there is a specific terminology in your community used for preterm birth, please use this or ask the group how they call it.

9. In your opinion, when is a baby born too soon? At what age/how many weeks or months?
   a. Do people expect most babies to be born after 9 months of pregnancy? How do you call this in the community?

10. What do people in your community think about babies who are born too soon / born before their due date (e.g. before 9 months)
    a. What are some of the common characteristics of a baby that is born too soon (PROBE: for small, low birthweight, has complications, delicate, unable to breastfeed, etc)
    b. How do people in the community discuss and react towards a woman who has a preterm labour and gives birth to a preterm baby,?
        i. Would most people find out about I or does a family not share this? If the family do not share the preterm birth, why is the reason?
        ii. Do people in your community worry about babies born too soon (preterm birth)? Why or why not?
    c. How do people in your community treat a woman and her family if there is a preterm birth?
    d. What do people in your community think are some of the reasons for having a preterm baby? What do they think causes a baby to be born before its due date?
    e. What do people in your community think are some of the ways to prevent preterm birth or early labour?
    f. Who do women who have a baby born too soon receive support from? Can they count on their husband, family, and neighbors for support? Where do they get advice?

Preterm birth care

In this section, we are aiming to understand how women in preterm labour are cared for and what women do if they go into labour before the baby is due.

11. What do pregnant women in your community do if they go into labour (for example start having labour pain or breaks water early), long before the baby is due to be born (before 9 months)?
    a. Would women in this case go to a health facility? Would they go to a different facility from where they would go for birth if it was not too soon?
        i. Is it easy to get to? How do they get there?
        ii. Who supports them to get there?
        iii. What have you heard about the care women receive there? Are women generally satisfied or not? Why?
    b. Are there some women who do not go to a health facility? Why do they not go to the facility? [Note: Probe for different challenges including availability of services, opening hours, location, lack of transport, cost, fear, perceptions of quality, lack of family support, other reasons.]

12. Have you heard of any medications that are given to women who have gone into labour much before the time when the baby is due? If so what medications?
    a. When and why are these medications given?

b. Do you think women in your community would take any medication to stop giving birth too soon? Why or why not? What might influence their decision?
c. The baby born too early may not be able to breathe on their own, as their lung may have not fully developed. Have you heard of any medicines to help a baby breath at birth (e.g. steroids)? What can you tell me about these medicines?
    i. Are these medicines available for women in your community? Where and how do women who go into labour before their due date get them?
    ii. Are there any women who may not get them? Why not?
d. What do you think might influence whether a woman with a high change of giving birth to a preterm infant would take any medication to help baby breathe better after birth?

Childbirth care

In this section, we would like to understand where women go for childbirth care and how they feel about the care they receive; we also want to know about women who give birth to a baby born too soon or born small.

13. Where do most women go for **childbirth care**?
    a. In the health facility? Which facility here is most used? Why do they choose this facility?
    b. Is it easy for women to get to the facility? [PROBE: for any challenges in going to the facility including transport, distance, cost, lack of family support]
    c. How do women feel about the care they receive in this facility?
    d. Are husbands and families invited to participate in caring for the newborn while the woman and baby are still in the health facility? What do they do and do not? Why/why not?
    e. How long after birth do a woman and her baby get discharged?
    f. Do most women feel that they are oriented on how to take care of their baby before being discharged to the home? What is the orientation given to them on discharge? Do they feel confident to go home with their baby? Why or why not?
    g. If women do not go to a health facility, who supports them with home birth?
    h. Do women in the community who delivered in the health facility receive appointment for follow-up visit? Do they go on the appointment date?
    i. Do women in the community receive any follow-up visit after the delivery at home?

14. Where do most women go for childbirth care if they are having a **preterm birth**?
    a. In the health facility? Which facility here is most used? How to they go to this facility? Why do they choose this facility?
    b. Is it easy for women to get to the facility? [PROBE: for any challenges in going to the facility including transport, distance, cost, lack of family support]
    c. How do women feel about the care they receive in this facility?
    d. Are husbands and families invited to participate in caring for the newborn baby while the woman and baby are still in the health facility? What are they allowed to do?
    e. How long after birth do a woman and her baby get discharged?
    f. Do most women feel that they are oriented on how to take care of their baby before being discharged to the home? What is the orientation given to them on discharge? Do they feel confident to go home with their baby? Why or why not?
    g. If women do not go to a health facility, who supports them with home birth?

15. If a woman has a home birth and has a baby that is born too soon, can you tell us what happens? (PROBE: does she get referred to a health facility? Does she usually get to the facility? If not, who takes care of the woman and baby?)

Newborn care

In this section, we would like to understand the care of newborns, including newborns who are born too soon.

16. Once a woman and baby are back in the home, who helps the woman to take care of the baby?

- Probe; Washing, feeding,
    - What are the challenges in taking care of the baby, what do they do to overcome the challenges?

17. Who do most women go to to get health advice on caring for their baby?

18. If a woman in your community gives birth to a baby born toon soon, what are some of the challenges she has?
    a. Who helps her to care for the preterm baby? How do they help?

19. What would you suggest be done if we wanted to convince people to adopt new behaviours for caring for newborns, and particularly newborns who are born too soon?

20. Who do you think we could involve from the community to help in improving how preterm babies are taken care of and to better support women to care for their babies? [probe; community stakeholder, health extension worker, health development army]
    a. How could we engage different community stakeholders and which should we engage?

Wrapping up

21. Are there any other issues I haven't brought up that you feel are important and you want to talk through?

Closing

Thank you for your time today. Your contributions will support the health services in better understanding how to improve maternal and newborn health, including the health of women who have preterm labour and their babies.

IDI: Formative assessment-In-depth interview with ANC Provider

#	*Item*	*Response options/ Codes*
	Identifiers	
1	*Participant ID*	
2	*Interview date*	
3	*Interviewer name*	
4	*Language of Interview*	
5	*Region*	
6	*Zone*	
7	*Woreda*	
8	*Health facility name*	
	Background information	
9	*What is your age in completed years?*	
10	*Sex*	
11	What is your marital status?	1. Single<br>2. Married/living together<br>3. Widowed<br>4. Separated<br>5. Other (specify)
12	How many children do you have? (If no child please enter 0)	
13	What is your professional background?	
14	What is your main role in this facility or community?	
15	How long have you worked in this facility or community?	

Purpose: Build rapport

- To begin, I'd like to get to know you a little bit more. Tell me, what is it like for you being a Health care worker?

Antenatal care and gestational age dating

- From your experience, how do you see the timing of ANC follow up?
    - When do most women seek antenatal care during pregnancy (how far along during pregnancy)?
    - Tell me the reasons why women don't come for antenatal care in the first trimester?
    - Do mothers pay for the antenatal care? How much do they pay? Do you think the payment affects utilization of antenatal care by mothers?
    - Do you have any suggestions about how the antenatal care could be improved?
- In your hospital/health facility, how do you currently date a woman's pregnancy / gestational age?
    - As a (midwife/nurse/doctor) what are some of the challenges you have in gestational age?
    - Can you tell me about the use of ultrasound to help assess gestational age?
- In your hospital/health facility, do women receive an ultrasound scan to help assess gestational age?
    - Do all women receive an ultrasound scan? who might not receive an ultrasound scan and why?
    - (If yes): can you tell me about this – when do women typically receive an ultrasound scan? How many ultrasound scans do women receive?
    - Who is responsible for giving the ultrasound scan, are they always available, what are the challenges with giving the ultrasound scan?
    - Do mothers pay for the ultrasound scan? How much do they pay? Do you think the payment affects utilization of antenatal care by mothers?
    - Do you have any suggestions about how the gestational age dating could be improved?

Preterm birth and danger signs

- In your opinion, when is a baby born too soon? At what age/how many weeks or months?
- In your opinion, is preterm birth important issue in your hospital/facility? Why or why not?
- As a (midwife/nurse/doctor), what do you do if/when a pregnant woman comes to the hospital/facility with signs of preterm labor?
- Have you heard of any ways to prevent preterm birth? If so, tell me?

Preterm/LBW infants feeding counselling

- How do you describe the counseling and education of preterm/LBW infants feeding
    - What information is given to the mother regarding breast feeding/formula feeding? When is this information provided?
    - Is counselling given to mothers about breastfeeding on a regular basis? If yes, when is this given and by whom?
    - Is a mother shown practically how to position and attach baby to the breast when she begins to breastfeed? Do you use any teaching material or job aids? Please tell me more…
    - What are the challenges you face with counselling on breastmilk feeding
- Have you ever experienced any religious or cultural practices that would negatively or positively influence a mother to practice exclusive breastfeeding? Can you give me an example?

ACS administration

- Can you tell me about medications to prevent preterm complications related to lung maturity? (e.g., antenatal corticosteroid/steroid/dexamethasone)?
    - Are they available for women in your hospital/facility? Where and how do they get them?
    - At what gestational age would you consider to give a woman antenatal corticosteroid/steroid/ dexamethasone?
    - Can you tell me factors you would consider to give a woman antenatal corticosteroid/steroid/ dexamethasone (probe: gestational age, membrane status, imminence of birth, signs of infection)
    - What do you think influences a woman to take any medication if she presents with preterm labor? (e.g., antenatal corticosteroid/steroid)?
- Would you feel confident prescribing antenatal corticosteroid/steroid/ dexamethasone for women in preterm labor? Why or why not? What might make you feel more confident? Less confident?
- I would like to know how you record data on ACS administration and what is the data used for. Could you please tell me about this? What are the challenges with the ACS data recording, reporting, and use?
- Do you have any suggestions about how the provision of antenatal corticosteroid/steroid/ dexamethasone for preterm birth could be improved?

IDI: Formative assessment-In-depth interview with Labor and delivery care provider

*# *	***Item***	***Response options/ Codes***
	Identifiers	
1	*Participant ID*	
2	*Interview date*	
3	*Interviewer name*	
4	*Language of Interview*	
5	*Region*	
6	*Zone*	
7	*Woreda*	
8	*Health facility name*	
	Background information	
9	*What is your age in completed years?*	
10	*Sex*	
11	What is your marital status?	1. Single<br>2. Married/living together<br>3. Widowed<br>4. Separated<br>5. Other (specify)
12	How many children do you have? (If no child please enter 0)	
13	What is your professional background?	
14	What is your main role in this facility or community?	
15	How long have you worked in this facility or community?	

Purpose: Build rapport

- To begin, I'd like to get to know you a little bit more. Tell me, what is it like for you being a health care worker?

Childbirth care and referral

- As a health care professional, what care do you routinely provide during childbirth? Please remember the last time you attended a birth and describe to me what you did from the start to end.
- Is the childbirth care you provide for low-birth weight and/or preterm infants different from the term infants? Please tell me more…
- Please tell me how babies are weighed in this facility
    - Are all babies weighed at birth in the facility?
    - How long after birth are they usually weighed?
    - What type of scales does the facility have? How often are the scales calibrated?
    - What is the practice of informing mothers about their newborn infant's weight?
    - What is the practice of recording newborn infant's weight?
    - What are the main reasons babies are not weighed for facility births?
    - What happens when a baby is identified with a low birth weight?
    - What is the lowest birth weight a baby can survive?
    - Do you have any suggestions about how the weighing of all babies and recording of the birth weight could be improved?
- Please tell me about referral care for newborn in your facility.
    - For what reasons would a newborn be referred to a higher facility?
    - How is the process? What are the available mechanisms for referral? How is information shared about the mother and baby referred? How well does the referral system work?
    - In situation where a mother and/or baby need to go to another health facility how would she go to the nearby health facility? alone? with her baby? What are the challenges with reaching a health facility??
    - Is the referral policy/guideline the same for low birth weight or early babies? How well is this policy/guideline followed?
    - Where are women referred if they have preterm labor or need treatment because a baby is born too soon?
    - How does a preterm mother get to the referral facility (e.g., transportation)? Who goes with her?
    - Do mothers pay for the referral care? How much do they pay? Do you think the payment affects acceptance of referral care by mothers? How?
    - Do you have any suggestions about how the referral care could be improved?
- Do you have linkage with health extension workers? How closely do you work with community health workers? Please tell me more…

Preterm birth and danger signs

- In your opinion, when is a baby born too soon? At what age/how many weeks or months?
- In your opinion, is preterm birth an important issue in your hospital/facility? Why or why not?
- As a (midwife/nurse/doctor), what do you do if/when a pregnant woman comes to the hospital/facility with signs of preterm contractions?
- Have you heard of any ways to prevent preterm birth? If so, tell me?

ACS administration

- Can you tell me about medications to prevent preterm complications related to lung maturity? (e.g., antenatal corticosteroid/steroid/ dexamethasone)?
    - Are they available for women in your hospital/facility? Where and how do they get them?
    - At what gestational age would you consider to give a woman antenatal corticosteroid/steroid/ dexamethasone?
    - Can you tell me factors you would consider to give a woman antenatal corticosteroid/steroid/ dexamethasone (probe: gestational age, membrane status, imminence of birth, signs of infection)
    - What do you think affects a woman to take any medication for preterm labor? (e.g., antenatal corticosteroid/steroid)?
- Would you feel confident prescribing antenatal corticosteroid/steroid for women in preterm labor? Why or why not? What might make you feel more confident? Less confident?
- I would like to know how you record data on ACS administration and what is the data used for. Could you please tell me about this? What are the challenges with the ACS data recording, reporting, and use?
- Do you have any suggestions about how the provision of antenatal corticosteroid/steroid for preterm birth could be improved?

Now I would like to learn more about the feeding support to preterm/low birth weight:

- From your experience, how do you describe the feeding of preterm/LBW infants?
    - What are the feeding options? Mechanisms?
    - Time of initiation of BF
    - How is the feeding of the baby determined? Who decides on the method of feeding options? Is this communicated with the mother/ family?
    - Is there a written policy, guideline or protocol on feeding of low birth weight and/or preterm infants that you follow? Please tell me more
    - If formula milk is given to babies, what are the reasons for giving formula milk? For how long formula milk is given?
    - Tell me about support you provide on breastfeeding of preterm/LBW infants
    - How do you compare exclusive breastmilk feeding to other feeding options (e.g., formula feeding) in terms of its effect on the survival, health, and nutritional outcomes of infants?
    - Do mothers pay for anything related to feeding of their low-birth weight and/or preterm infant (Cup, NG tube, breast pump etc…)? How much? Do you think the payment affects utilization of feeding support service by other mothers?
    - What are the challenges you face with breastfeeding/expressed breast milk feeding of preterm/LBW infants?
    - What should be done to overcome this challenge or solve the problem?
- How do you describe the counseling and education of preterm/LBW infants feeding
    - What information is given to the mother regarding breast feeding/formula feeding? When is this information provided?
    - Is counselling given to every mother with preterm/LBW on breastfeeding? If yes, when is this given? By who?
    - Is a mother shown practically how to position and attach baby to the breast when she begins to breastfeed? Do you use any teaching material or job aids? Please tell me more…
    - Are mothers taught how to express breast milk? Who teaches them?
    - What are the challenges you face with counselling and supporting mothers with breastmilk feeding (either expressed by tube or cub or direct breastfeeding)? (Time, workload, number of staff)
    - What should be done to overcome this challenge or solve the problem?
- For babies who cannot directly feed from the breast, it is recommended for mother to express breast milk.

  - Is there a space where mothers can privately express/pump breast milk?
  - Do you think the space would allow to do that? Why or why not? What do you think needs to be done so that mothers can breastfeed their babies?
  - Do they have access to breast pump?
  - What about handwashing facility?
  - Where do mothers store the expressed breastmilk? A container to store the expressed breastmilk? Feeding cup? Refrigerator?
  - What do you think needs to be done so that mothers can express breast milk?

- Do you think mothers' and families' needs and preferences in feeding of their low birth weight and/or preterm infant are met? What should be done to address their needs and preferences?
- From your experience, have you ever encountered any religious or cultural practices that would negatively or positively influence a mother to practice exclusive breastfeeding? Can you give me an example?
- I would like to know how the data on breastmilk feeding of low birth weight and/or preterm infants is recorded and what is the data used for. Could you please tell me about this? What are the challenges with the feeding data recording, reporting, and use?

Discharge process

- How long do mothers with preterm/LBW usually stay in the labor and delivery unit?
- Can you tell me about the discharge process? What criteria do you use to discharge low-birth weight and/or preterm newborns?
  - Counselling of mothers and family members before discharge
  - Checking if the child is breastfeeding
  - Next appointment date
- What challenges do you face in the discharge process of low birth weight and/or preterm infants?
- Do you have any suggestions about how the discharge care could be improved?

Postnatal care

- Now I would like to learn about your postnatal care experience. Do you provide postnatal care for low birth weight and/or preterm infants? If yes, probe the following
  - What do you provide during the postnatal care visit? How many postnatal care visits are low birth weight and/or preterm infants expected to have?
  - What are the challenges you face in postnatal care provision?
- Do mothers pay for the postnatal care? How much do they pay? How do you think the payment affects utilization of postnatal care by mothers?
- Can you tell me the support (if any) a women received after discharge (at home)
  - Do women and newborns receive home visits after discharge?
  - Does a community health worker visit small or early babies after discharge at home? How long after discharge? What is done during the visit?
  - What are the main reasons families may not receive a visit?
- Do you have any suggestions about how the postnatal care could be improved?

Wrapping up

- Are there any other issues I haven't brought up that you feel are important and you want to talk through?

IDI: Formative assessment-In-depth interview with health extension workers (HEWs)

#	Item	Response options/ Codes
	**Identifiers**	
1	Participant ID	
2	Interview date	
3	Interviewer name	
4	Language of Interview	
5	Region	
6	Zone	
7	Woreda	
8	Health facility name	
	**Background information**	
9	What is your age in completed years?	
10	Sex	
11	What is your marital status?	1. Single<br>2. Married/living together<br>3. Widowed<br>4. Separated<br>5. Other (specify)
12	How many children do you have? (If no child please enter 0)	
13	What is your professional background?	
14	What is your main role in this facility or community?	
15	How long have you worked in this facility or community?	

Purpose: Build rapport

- To begin, I'd like to get to know you a little bit more. Tell me, what is it like for you being a health care worker?

Sick and small newborn care and feeding practices

- Can you tell me about the routine care you provide to low birth weight and/or preterm infants at your facility (neonatal unit)? *Please probe on the below, allow the respondent to describe the care processes*
    - Feeding support to the infant
    - Skin-to-skin care
    - Medications
    - Oxygen/CPAP support
    - Referral care
    - Care for the mothers
- What problems can babies born too early or with low birth weight have?

Now I would like to learn more about the feeding support to the infant:

- From your experience, how do you describe the feeding of preterm/LBW infants?
    - What are the feeding options? Mechanisms?
    - Time of initiation of BF
    - How is the feeding of the baby determined? Who decides on the method of feeding options? Is this communicated with the mother/ family?
    - Is there a written policy, guideline or protocol on feeding of low birth weight and/or preterm infants that you follow? Please tell me more
    - If formula milk is given to babies in the NICU, what are the reasons for giving formula milk? For how long formula milk is given?
    - Tell me about breastfeeding of preterm/LBW infants
    - How do you compare exclusive breastmilk feeding to other feeding options (e.g., formula feeding) in terms of its effect on the survival, health, and nutritional outcomes of your infant?
    - Do mothers pay for anything related to feeding of their low-birth weight and/or preterm infant? How much? Do you think the payment affect utilization of feeding support service by other mothers?
    - What are the challenges you face with feeding of preterm/LBW infants?
    - What should be done to overcome this challenge or solve the problem?
- How do you describe the counseling and education of preterm/LBW infants feeding
    - What information is given to the mother regarding breast feeding? When is this information provided?
    - What information is given to the mother regarding formula feeding? When is this information provided?
    - Is counselling given to mothers about breastfeeding on a regular basis? If yes, when is this given? By who?
    - Is a mother shown practically how to position and attach baby at the breast when she begins to breastfeed? Do you use any teaching material or job aids? Please tell me more…
    - Are mothers taught how to express breast milk? Who teaches them?
    - What are the challenges you face with counselling and supporting mothers with breastmilk feeding (either expressed

by tube or cub or direct breastfeeding)? (Time, workload, number of staff)

- What should be done to overcome this challenge or solve the problem?

- For babies who cannot directly feed from the breast, it is recommended for mother to express breast milk.
    - Is there a space where mothers can privately express/pump breast milk?
    - Do you think the space would allow to do that? Why or why not? What do you think needs to be done so that mothers can breastfeed their babies?
    - Do they have access to breast pump?
    - What about handwashing facility?
    - Where do mothers store the expressed breastmilk? A container to store the expressed breastmilk? Feeding cup? Refrigerator?
    - What do you think needs to be done so that mothers can express breast milk?
- What do you think is the most difficult challenge/problem mothers and families face regarding feeding when their newborn is in the NICU?
- What should be done to overcome this challenge or solve the problem?
- Do you think mothers' and families' needs and preferences in feeding of their low birth weight and/or preterm infant are met? What should be done to address their needs and preferences?
- Have you ever experienced any religious or cultural practices that would negatively or positively influence a mother to practice exclusive breastfeeding? Can you give me an example?
- I would like to know how the data on breastmilk feeding of low birth weight and/or preterm infants is recorded and what is the data used for. Could you please tell me about this? What are the challenges with the feeding data recording, reporting, and use?

NICU space and HR

- I do not know much about the neonatal care unit of your facility. Can you describe the space to me?
    - What do you like/dislike about the space?
    - How would you describe the cleanliness? Ventilation? Temperature? Privacy?
    - Is the mother allowed in the NICU? If yes, for how long is she allowed with the baby? When she is in the NICU what does she do?
- Tell me about your experience as small and sick newborn care provider
    - Are you trained to provide care for the sick and small newborns? To provide support to mothers of low-birth weight infants on breastmilk feeding? Could you please tell me more about the training you received?
    - Do you receive regular mentorship support on care for the sick and small newborns? To provide support to mothers of low-birth weight infants on breastmilk feeding? Could you please tell me more about the mentorship you received?
    - What kind of support do you think needs to be available to support breastfeeding of Low birth weight and preterm babies?
    - How confident do you feel in your knowledge and skills in provision of care for small and sick newborns? In provision of counselling and support on feeding of low birth weight and/or preterm infants?
    - Do you think nurses in the NICU currently support and promote breastfeeding or breast milk feeding practice? Why/why not? Can you give me an example?
    - Do you think physicians in the NICU currently support and promote breastfeeding or breast milk feeding practice? Why/why not? Can you give me an example?
    - Tell me about the staff at the newborn care unit. How many are they? How many of them are trained to provide care

for the sick and small newborns? To provide support to mothers of low-birth weight infants on breastmilk feeding?

- Please tell me about engagement of mothers and families in the neonatal care
    - Are mothers and family members involved in the care of the care of their low-birth weight and/or preterm newborns?
    - How do you wish to engage them?
- Do mothers pay for the neonatal care services? How much? Do you think the payment affect utilization of neonatal care by other mothers?
- Please tell me about referral care for newborn in your facility.
    - For what reasons would a newborn be referred to a higher facility?
    - How is the process? What are the available mechanisms for referral? How is information shared about the mother and baby referred? How well does the referral system work?
    - In situation where a mother and/or baby need to go to another health facility how would she go to the nearby health facility? alone? with her baby? What are the challenges with reaching a health facility??
    - Is the referral policy/guideline the same for low birth weight or early babies? How well is this policy/guideline followed?
    - Where are women referred if they have preterm labor or need treatment because a baby is born too soon?
    - How does a preterm mother get to this place (e.g., transportation)? Who goes with her?
    - Do mothers pay for the referral care? How much do they pay? Do you think the payment affect acceptance of referral care by mothers? How?
    - Do you have any suggestions about how the referral care could be improved?
- Do you have linkage with community health workers? How closely do you work with community health workers? Please tell me more…

Discharge process

- How long do mothers usually stay in the neonatal care unit or at the health facility?
- Can you tell me about the discharge process? What criteria do you use to discharge low-birth weight and/or preterm newborns?
- What challenges do you face in the discharge process of low birth weight and/or preterm infants?
- Do you have any suggestions about how the discharge care could be improved?

Wrapping up

- Are there any other issues I haven't brought up that you feel are important and you want to talk through?

IDI: Formative assessment-In-depth interview guide for maternal health stakeholder

#	Item	Response options/ Codes
	Identifiers	
1	Participant ID	
2	Interview date	
3	Interviewer name	
4	Language of Interview	
5	Region	
6	Name of the organization	
	Background information	
7	What is your age in completed years?	
8	Sex	
9	What is your marital status?	1. Single<br>**2.** Married/living together<br>**3.** Widowed<br>**4.** Separated<br>**5. Other**
10	What is your professional background?	
11	What is your main role in this organization?	
12	How long have you worked in this organization?	

Purpose: Build rapport

- To begin, I'd like to get to know you a little bit more. Tell me, what is it like for you being in your current role?

Antenatal care and gestational age dating

- From your experience, when do most women seek antenatal care during pregnancy (how far along during pregnancy)? Rural women? Urban women?
- Can you tell me the reasons why women do not come for antenatal care in the first trimester?
    - *In the IDIs conducted with health care providers and community, we identified some of the barriers for ANC service. These included feeling shy to go for ANC early/before the woman's pregnancy shows, lack of confidence on the HCP, fear of mistreatment by HCPs, unplanned/out of wedlock pregnancy, lack of transportation, lack of money, long waiting time, distance from the health facility and lack of supplies in some of the health facilities.* Do you believe these barriers affect women utilizing ANC service? Can you elaborate further?
    - Do you have any suggestions about how the antenatal care could be improved?
- In hospitals/other health facility, how is gestational age assessed?
    - What are some of the challenges in assessing Gestational age?
- Can you tell me about the use of ultrasound to determine gestational age? (policy/guideline, availability of equipment/other supplies/ trained human resource)
- Is there a data recording system related to gestational age at the ANC. Please tell me more about the system. If no, why?
    - *In the IDIs conducted with health care providers and community, we identified some of the barriers for ultrasound service. These included unavailability and non-functional ultrasound machine, lack/shortage of trained health care providers, long waiting time, misconceptions about ultrasound scan by mothers.* Do you think these barriers are also common elsewhere (other woredas, region wide or nationally)?
    - Do you have suggestions on how access to obstetric ultrasound scan care could be improved?
- Can you tell me about the overall financing of ANC? (national/health facility level)
    - *From the IDIs we conducted with healthcare providers, mothers and community members we learned that pregnant mothers incur some cost for ANC service (lab test, medication) and ultrasound scan service? Do you think this is common elsewhere (other hospitals, region wide or nationally)?*

  - Do you think payment for medication, laboratory test, registration card affects the ANC services utilization?
  - Do you think payment for Ultrasound scan affects the service utilization? How?
  - Do you have any suggestions on what should be done to ensure all mothers can access comprehensive ANC including ultrasound scan without financial hardship?

ACS administration

- Please tell me about antenatal corticosteroid/steroid use in Ethiopia?
  - How do you describe the current policy/recommendation regarding antenatal corticosteroid/steroid in Ethiopia?
  - Can you tell me about the gestational age when antenatal corticosteroid/steroid is recommended?
  - What are the barriers/challenges and facilitators in antenatal corticosteroid/steroid use in Ethiopia? (human resource, drug availability, payment for service)
  - *In the IDIs we conducted with health care providers and community, we identified some of the barriers for ACS use. These included lack of awareness/knowledge by both the HCPs and community, lack of adequate training of HCPs, inability to date gestational aga, and unavailability of ACS drugs.* Do you think these barriers are common elsewhere (other hospitals, region wide, or nationally?
  - Do you have any suggestions on what should be done to ensure all eligible mothers access quality and safe ACS?
- Can you please tell me about a data recording system related to ACS administration and what is the data used for. Could you please tell me about this? What are the challenges with the ACS data recording, reporting, and use?

Childbirth care and referral

- Can you please describe the current recommendation and practice about childbirth care service for low-birth weight and/or preterm infants? Is it different from the term infants? Please tell me more…
- Please tell me how babies are weighed
  - Guideline on birth weight measurement at facility and community?
  - How do you evaluate the practice of recording newborn infant's weight?
  - What are the main reasons babies born in health facility might not be weighed?
  - Do you have any suggestions about how the weighing of all babies and recording of the birth weight could be improved?
- Please tell me about the referral process for newborn care?
  - What are the reasons a newborn would be referred to a higher facility? How well does the referral system work?
  - Is the referral policy the same for low birth weight or early babies? How well is this policy followed?
  - Where are women referred if they have preterm labor or need treatment because a baby is born too soon?
  - How does she get to the referral facility (e.g., Ambulance)?
  - *From the IDIs conducted with health care providers and community we learned that families pay for fuel and driver per-diem to use the ambulance service. Is this a common practice in other sites, region wide or nationally? Why?* Do you think the payment affects acceptance of referral care by mothers? How?
  - Do you have any suggestions about how referral care could be improved?

Discharge process

- Can you tell me about the health care service provision linkage after preterm/LBW infants are discharged to home?
- Can you tell me about the discharge process for low-birth weight and preterm newborns?
  - Criteria for discharge, challenges?
  - *In the IDIs conducted with health care providers and community, we learned that there is a practice of early discharge of preterm/LBWs before they start breastfeeding and gain weight. Also, mothers are not adequately counselled prior to discharge? Do you think these challenges are common elsewhere (other hospitals, region wide or nationally)? Why?*
- Is there a regionally or nationally approved guideline with defined set of criteria to the discharge of preterm/LBW infants?
- Do you have any suggestions about how the discharge care could be improved?

Postnatal care

- How do you describe the postnatal care service? Is PNC different for preterm/LBW? How?
  - Payment for the postnatal care and its effect on utilization?
  - Home visits after discharge? What are the main reasons families may not receive a PNC home visit?
    - *From the IDIs conducted we have learned that during PNC visits do happen rarely or not happen at all. Do you think this is the case elsewhere (other Woredas, region wide or nationally)? Why?*

        - *From the IDIs conducted we have learned the neonatal care given at the community level (home visits and health post services) is interrupted due to shortage of supplies, equipment and security issue. Do you think this is the case elsewhere (other Woredas, region wide or nationally)? Why?*
- Do you have any suggestions about how the postnatal care could be improved?

Policy and program

- How do you evaluate engagement, commitment, accountability of leadership regarding small and sick newborn care? (Probe: leadership for preterm/LBW breast feeding, ANC, obstetric ultrasound scan, ACS)
    - Is the care/service given to preterm/low birth weight infants a priority? How and why?
    - How do you describe the policy or leadership level barriers and facilitators regarding small and sick newborn care? (for preterm/LBW breast feeding, ANC, obstetric ultrasound scan, ACS)
- Can you tell me about the level of stakeholders' engagement and coordination to support small and sick newborn care? (Probe: for preterm/LBW breast feeding, ANC, obstetric ultrasound scan, ACS)
- What are the barriers and facilitators of supply chain management (Drug and equipment) for small and sick newborn care? (Prob: barriers and facilitators for preterm/LBW breast feeding, ANC, obstetric ultrasound scan, ACS)?
- What do you recommend to mitigate system level barriers for small and sick newborn care? (Probe: Finance, report, supply chain management for preterm/LBW breast feeding, ANC, obstetric ultrasound scan, ACS)

Wrapping up

- Are there any other issues I haven't brought up that you feel are important and you want to talk through?

IDI: Formative assessment-In-depth interview guide for SSNC stakeholder

#	Item	Response options/ Codes
	Identifiers	
1	Participant ID	
2	Interview date	
3	Interviewer name	
4	Language of Interview	
5	Region	
6	Name of the organization	
	Background information	
7	What is your age in completed years?	
8	Sex	
9	What is your marital status?	6. Single<br>**7.** Married/living together<br>**8.** Widowed<br>**9.** Separated<br>**10. Other**
10	What is your professional background?	
11	What is your main role in this organization?	
12	How long have you worked in this organization?	

Purpose: Build rapport

- To begin, I'd like to get to know you a little bit more. Tell me, what is it like for you being in your current role?

Sick and small newborn care and feeding practices

- Can you describe the NICU service in Ethiopia (Oromia, Batu, Meki hospitals)?
    - Are they equipped with the necessary logistics
    - How would you describe the space adequacy? Cleanliness? Ventilation? Temperature? Privacy?
        - *In the IDIs conducted with health care providers and community, we identified that there is lack of space and essential facilities such as functional hand washing facility, place for mothers/families to eat, sleep or rest. Do you think this is a common challenge in other hospitals providing NICU service, region wide, nationally? Why?*
    - How do you describe the engagement of mothers (families) in SSNC in the NICUs?
        - *From the IDIs we conducted we learned that hospitals allow mothers in the NICU only for brief breast-feeding session. Is this is a common practice in other hospitals providing NICU service, region wide, nationally? Why?*
        - *Do you think limiting the time mothers (and families) spend providing care to their sick newborns in the NICU affect the health outcomes of the Newbon? Why/Why not?*
        - *From the IDIs we conducted we learned that mothers face mistreatment from some of the health care providers at the NICU. Do you think this is the practice in other hospitals, region wide, nationally? Why?*
        - What do you suggest should be done to improve the engagement of mothers (families) in SSNC and improve their experience?
- Tell me about small and sick newborn care providers capacity building
    - Are HCP skilled to provide care for the sick and small newborns? Please tell me more
        - *From the IDIs we conducted we learned that the NICU service is affected by lack of training, shortage of staff, lack of mentorship, and high workload? Do you agree with this? Are these challenges common in other hospitals, regionwide or nationally? Why?*
        - *What do you suggest should be done to improve the skills and motivation of SSNC providers?*

- Can you please tell me about the overall financing/budgeting related to neonatal care service? (national/regional/health facility level)
    - *From the IDIs we conducted with the health care providers and mothers/ fathers, learned that newborns are not covered by CBHI and hence in the last year or so hospitals started to make families pay for NICU service. Do you think this is the practice in other hospitals, region wide, nationally? Why did hospitals introduce payment for NICU service?*
    - Do you think the payment for NICU services affect utilization of neonatal care service?
- Please tell me about the referral process for newborn care?
    - What are the reasons a newborn would be referred to a higher facility? How well does the referral system work?
    - Is the referral policy the same for low birth weight or early babies? How well is this policy followed?
    - Where are women referred if they have preterm labor or need treatment because a baby is born too soon?
    - How does she get to the referral facility (e.g., Ambulance)?
    - Do mothers pay for the referral care? How much do they pay? Do you think the payment affects acceptance of referral care by mothers? How?
        - *From the IDIs conducted with health care providers and community we learned that families pay for fuel and driver per-diem to use the ambulance service. Is this a common practice in other sites, region wide or nationally? Why?*
    - Will this affect access to referral care for families who couldn't afford to pay?
    - How do you think the ambulance service can be accessed by families who couldn't afford to pay?
- Now I would like to learn more about the feeding support to the preterm/low birth weight infant:
    - Can you please tell me about the existing policy, guideline or protocol on feeding of low birth weight and/or preterm? Please tell me more
    - How do you describe the counseling practice given to mothers about breastfeeding of preterm and/or low birth weight newborns? How is it given? When is this given? By who?
    - Do you think nurses and physicians (Ask separately for both professions) in the NICU currently support and promote breastfeeding or breast milk feeding practice? Why/why not? Can you give me an example?
    - Do you think health care providers have the required skills to educate and practically support mothers on breastfeeding positioning, attachment, and express breast milk to feed preterm and/or low birth weight newborns?
    - Do health care providers use any teaching material or job aids to counsel or support mothers and families on breastfeeding of preterm and/or low birth weight newborns? Please tell me more…
    - From your experience could you please describe the challenges health care providers face with counselling and supporting mothers on breast milk feeding of preterm and/or low birth weight newborns (either expressed by tube or cub or direct breastfeeding)? (Time, workload, number of staff)
        - *In the IDIs conducted with health care providers and community, we identified that lack of teaching aids, staff negligence, and lack of training and mentorship as barriers to breast feeding counseling and support to preterm and/or low birth weight infants? Do you think these challenges are common elsewhere (other hospitals, region wide or nationally)? Why?*
    - What kind of support do you think needs to be available to support breastfeeding of preterm and/or low birth weight infants?
    - How do you compare exclusive breast milk feeding to other feeding options (e.g., formula feeding) in terms of its effect on the survival, health, and nutritional outcomes of your infant?
        - If formula milk is given to babies, what are the reasons for giving formula milk?
    - For babies who cannot directly feed from the breast, what is the recommendation for feeding preterm and/or low-birth-weight newborns?
        - Is there a space where mothers can privately express/pump breast milk?
        - Do they have access to breast pump?
        - What about proper hand washing facility?
        - Where do mothers store the expressed breast milk? Do they have access to a container to store the expressed breast milk? Feeding cup? Refrigerator?
        - What do you think needs to be done so that mothers can express breast milk, store and feed their preterm and/or low birth weight infants?
    - What do you think is the most difficult challenge/problem mothers and families face regarding feeding when their new-born is in the NICU?
    - What should be done to overcome this challenge or solve the problem?
    - Do mothers pay for anything related to feeding of their low-birth weight and/or preterm infant? How much? Do you think the payment affect utilization of feeding support service by other mothers?
        - *From the IDIs we had with families and healthcare providers we learned that mothers pay for breast pump, NG tube, and cup to express and feed their preterm and/or low birth weight infants in the NICU. Do you think this is the case elsewhere (other hospitals, region wide or nationally)? Why?*
        - Do you think this have an effect on the mother? How?

        - What do you suggest should be done to enable all preterm and/or low birth weight infants in the NICU are fed on expressed breast milk without financial burden on the families?
- Do you think mothers' and families' needs and preferences in feeding of their low birth weight and/or preterm infant are met? What should be done to address their needs and preferences?
- Can you tell me any religious or cultural practices that would negatively or positively influence a mother to practice exclusive breastfeeding? Can you give me an example?
    - *In the IDIs conducted with health care providers and community, we identified that it is common to provide animal milk to newborns, practice prelacteal feeding, discard colostrum and family members discourage mothers to breastfeeding their preterm/LBWs because of their low survival chance. Do you think this is the case elsewhere (other hospitals, region wide or nationally)? Why?*
- I would like to know how the data on breast milk feeding of low birth weight and/or preterm infants is recorded and what the data is used for. Could you please tell me about this? What are the challenges with the feeding data recording, reporting, and use?
    - How is the data recording system related to gestational age at the NICU? Please tell me more about the system. If not, why?

Discharge process

- Can you tell me about the health care service provision linkage after preterm/LBW are discharged to home?
- Can you tell me about the discharge process for low-birth weight and preterm newborns? (Criteria for discharge, challenges)?
    - *In the IDIs conducted with health care providers and community, we learned that there is a practice of early discharge of preterm/LBWs before they start breastfeeding and gain weight. The is also lack of counselling prior to discharge? Do you think these challenges are common elsewhere (other hospitals, region wide or nationally)? Why?*
- Is there a regionally or nationally approved guideline with defined set of criteria to the discharge of preterm/LBW infants?
- Do you have any suggestions about how the discharge care could be improved?

Postnatal care

- How do you describe the postnatal care service? Is PNC different for preterm/LBW? How?
    - Payment for the postnatal care and its effect on utilization?
    - Home visits after discharge? What are the main reasons families may not receive a PNC home visit?
        - *From the IDIs conducted we have learned that during PNC visits do happen rarely or not happen at all. Do you think this is the case elsewhere (other Woredas, region wide or nationally)? Why?*
        - *From the IDIs conducted we have learned the neonatal care given at the community level (home visits and health post services) is interrupted due to shortage of supplies, equipment and security issue. Do you think this is the case elsewhere (other Woredas, region wide or nationally)? Why?*
- Do you have any suggestions about how the postnatal care could be improved?

Policy and program

- How do you evaluate engagement, commitment, and accountability of leadership regarding small and sick newborn care? (Probe: leadership for SSNC, preterm/LBW breast feeding)
    - Is the care/service given to preterm/low birth weight infants a priority? How and why?
    - How do you describe the policy or leadership level barriers and facilitators regarding small and sick newborn care? (SSNC, preterm/LBW breast feeding)
- Can you tell me about the level of stakeholders' engagement and coordination to support small and sick newborn care? (Probe: SNNC, preterm/LBW breast feeding)
- What are the barriers and facilitators of supply chain management (Drug and equipment) for small and sick newborn care? (Prob: barriers and facilitators for SSNC and breast milk feeding)?
- What do you recommend to mitigate system level barriers for small and sick newborn care? (Probe: Finance, report, supply chain management for SSNC and breast milk feeding)

Wrapping up

- Are there any other issues I haven't brought up that you feel are important and you want to talk through?

IDI: Formative assessment-In-depth interview guide for nutrition stakeholder

#	Item	Response options/ Codes
	Identifiers	
1	Participant ID	
2	Interview date	
3	Interviewer name	
4	Language of Interview	
5	Region	
6	Name of the organization	
	Background information	
7	What is your age in completed years?	
8	Sex	
9	What is your marital status?	11. Single<br>**12.** Married/living together<br>**13.** Widowed<br>**14.** Separated<br>**15. Other**
10	What is your professional background?	
11	What is your main role in this organization?	
12	How long have you worked in this organization?	

Purpose: Build rapport

- To begin with, I'd like to get to know you a little bit more. Tell me, what is it like for you being in your current role?

Feeding preterm and/or low birth weight infants

- Now I would like to learn more about the feeding support to the preterm/low birth weight infant:
  - Can you please tell me about the existing policy, guideline or protocol on feeding of low birth weight and/or preterm? Please tell me more
  - How do you describe the counseling practice given to mothers about breastfeeding of preterm and/or low birth weight newborns? How is it given? When is this given? By who?
  - Do you think nurses and physicians (Ask separately for both professions) in the NICU currently support and promote breastfeeding or breast milk feeding practice? Why/why not? Can you give me an example?
  - Do you think health care providers have the required skills to educate and practically support mothers on breastfeeding positioning, attachment, and express breast milk to feed preterm and/or low birth weight newborns?
  - Do health care providers use any teaching material or job aids to counsel or support mothers and families on breastfeeding of preterm and/or low birth weight newborns? Please tell me more…
  - From your experience could you please describe the challenges health care providers face with counselling and supporting mothers on breast milk feeding of preterm and/or low birth weight newborns (either expressed by tube or cub or direct breastfeeding)? (Time, workload, number of staff)
    - *In the IDIs conducted with health care providers and community, we identified that lack of teaching aids, staff negligence, and lack of training and mentorship as barriers to breast feeding counseling and support to preterm and/or low birth weight infants? Do you think these challenges are common elsewhere (other hospitals, region wide or nationally)? Why?*
  - What kind of support do you think needs to be available to support breastfeeding of preterm and/or low birth weight infants?
  - How do you compare exclusive breast milk feeding to other feeding options (e.g., formula feeding) in terms of its effect on the survival, health, and nutritional outcomes of your infant?

        - If formula milk is given to babies, what are the reasons for giving formula milk?
    - For babies who cannot directly feed from the breast, what is the recommendation for feeding preterm and/or low-birth-weight newborns?
        - Is there a space where mothers can privately express/pump breast milk?
        - Do they have access to breast pump?
        - What about proper hand washing facility?
        - Where do mothers store the expressed breast milk? Do they have access to a container to store the expressed breast milk? Feeding cup? Refrigerator?
        - What do you think needs to be done so that mothers can express breast milk, store and feed their preterm and/or low birth weight infants?
    - What do you think is the most difficult challenge/problem mothers and families face regarding feeding when their new-born is in the NICU?
    - What should be done to overcome this challenge or solve the problem?
    - Do mothers pay for anything related to feeding of their low-birth weight and/or preterm infant? How much? Do you think the payment affect utilization of feeding support service by other mothers?
        - *From the IDIs we had with families and healthcare providers we learned that mothers pay for breast pump, NG tube, and cup to express and feed their preterm and/or low birth weight infants in the NICU. Do you think this is the case elsewhere (other hospitals, region wide or nationally)? Why?*
        - Do you think this have an effect on the mother? How?
        - What do you suggest should be done to enable all preterm and/or low birth weight infants in the NICU are fed on expressed breast milk without financial burden on the families?
- Do you think mothers' and families' needs and preferences in feeding of their low birth weight and/or preterm infant are met? What should be done to address their needs and preferences?
- Can you tell me any religious or cultural practices that would negatively or positively influence a mother to practice exclusive breastfeeding? Can you give me an example?
    - *In the IDIs conducted with health care providers and community, we identified that it is common to provide animal milk to newborns, practice prelacteal feeding, discard colostrum and family members discourage mothers to breastfeeding their preterm/LBWs because of their low survival chance. Do you think this is the case elsewhere (other hospitals, region wide or nationally)? Why?*
- I would like to know how the data on breast milk feeding of low birth weight and/or preterm infants is recorded and what the data is used for. Could you please tell me about this? What are the challenges with the feeding data recording, reporting, and use?
    - How is the data recording system related to gestational age at the NICU? Please tell me more about the system. If not, why?

Policy and program

- How do you evaluate engagement, commitment, and accountability of leadership regarding small and sick newborn care? (Probe: leadership for preterm/LBW breast feeding)
    - Is the care/service given to preterm/low birth weight infants a priority? How and why?
    - How do you describe the policy or leadership level barriers and facilitators regarding small and sick newborn care? (preterm/LBW breast feeding)
- Can you tell me about the level of stakeholders' engagement and coordination to support small and sick newborn care? (Probe: for preterm/LBW breast feeding)
- What are the barriers and facilitators of supply chain management (Drug and equipment) for small and sick newborn care? (Prob: barriers and facilitators for breast milk feeding)?
- What do you recommend to mitigate system level barriers for small and sick newborn care? (Probe: Finance, report, supply chain management of breast milk feeding)

Wrapping up

- Are there any other issues I haven't brought up that you feel are important and you want to talk through?
