# Supplementary material for: Applying the socioecological model to examine the beliefs, perceptions and attitudes surrounding preterm birth in Ethiopia: a qualitative study
Source: BMJ Open. 2026 Feb 6;16(2):e093030. doi: 10.1136/bmjopen-2024-093030 (PMC12887518; doi:10.1136/bmjopen-2024-093030)
Supplement: Supplementary file 2 [file bmjopen-2024-093030.reviewer_comments.pdf]

PEER REVIEW HISTORY

BMJ Open publishes all reviews undertaken for accepted manuscripts. Reviewers are asked to complete a checklist review form and are provided with free text boxes to elaborate on their assessment. These free text comments are reproduced below.

ARTICLE DETAILS

Title (Provisional)

Applying the socioecological model to examine the beliefs, perceptions, and attitudes surrounding preterm birth in Ethiopia: A qualitative study

Authors

Estifanos, Abiy Seifu; Gelaw, Meron Addis; Getachew, Hewan; Ireso, Beyene Roba; Dimtse, Asrat; Metaferia, Gesit; Woldehawariat, Tequam Debebe; Walelegn, Miraf; Magge, Hema; Roro, Meselech Assegid; Gobena, Rediet Gezahegn; Nigatu, Yakob Desalegn; Mengistu, Yalemwork; Shikur, Bilal; Demissew, Rahel; Beyene , Selemawit Asfaw; Tumilowicz, Alison

---

VERSION 1 - REVIEW

**Reviewer**	**1**
**Name**	**Silva, Thais**
**Affiliation**	**State University of Campinas, Obstetric and Gynecology**
**Date**	**04-May-2025**
**COI**	**None**

This is a very interesting analysis, since most part of prematurity worldwide comes from low-income countries, which makes urgent to understand possible cultural and educational barriers not only for parents, but also for the community and caregivers. Without such specific characteristics related to social relations, it is almost impractical to introduce preventable actions in different populations waiting for the same reductions in prematurity rate. Therefore, this qualitative analysis can be helpful to clarify some aspects to guide future target interventions for this population. However, I would like to have some clarifications in order:

1) How was defined the total sample? Which criteria were used to select participants using the hospital registers? The investigator included all preterm birth registers? How was the recruitment for focus group discussions?

2) The semi-structured guides used to conduct the in-depth interviews and the focus group discussions should be available on supplementary information. I would like to evaluate the questions, points and addressed issues contained on these guides.

3) Table 1: Line 54 Wouldn't be better also use the term “women” despite “mother” as done in Line 51? The manuscript do not include how many interviewed women experienced the loss of their child. Would be interesting to have this information.

4) Line 374: It was difficult to understand if the preterm delivery rate on health centers is small or if the health care providers do not understand this rate clearly. It should be clearer. If the preterm delivery rate is really small, why do not recruit professionals from health centers with a higher number of preterm births on that region?

5) The discussion section do not include strengths and limitations. Please, include this information.

Reviewer 2

Name Ekhaguere, Osayame

Affiliation Indiana University Department of Pediatrics, Neonatal-Perinatal Medicine

Date 05-Oct-2025

COI None

Thank you fro the opportunity to review this manuscript. The topic is very relevant to understanding parental and societal perceptions of preterm births. The justification for conducting the study are strong. The methods could be improved by providing information on current policies defining “preterm vs abortion" in Ethiopia and describing the clinical capacity of the health facilities that cater to the study community. Only by understanding the context in which the study was conducted can the results be interpreted and understood completely. While the results using the SEM is good, the results would be been more organized if theme under each branch of the SEM were provided. Finally, are most important, the conclusions of the study do not neccessarily reflect the findings of the study. Specifically, there is nothing in the results that suggests home healthcare practices, the healthcare-seeking behavior of families are influenced if the baby is premature or that healthcare services at facilities, and the motivation and will of healthcare providers to deliver these services to preterm are impeded by their perception of preterm birth. They may be influenced by the availability to provide these services, but this was not explored in the study so the conclusion cannot be made.

See below specific comments.

Below are my comments regarding the different sections of the write up.

Title: I think the title is over sensationalized, especially given the statement was not universally echoed by the study participants. One quote from a stake holder (not even an

individual who has experienced the personal loss of a preterm infant) should not dictate the title of a publication.

Abstract:

Methods: The way Batu and Meki are described as sites makes it look like they are institutions in which the study was conducted. Please consider replacing site with community.

Results: It seems there is a typographical error in the first statement of the result section.

“Giving {birth seems to be missing} to a preterm birth is often associated with fear, stress, unhappiness, concern, and 46 worry”.

I would have expected the results section to have a major theme for each level of the ecological model.

Conclusion: The conclusion should reflect the results that are shown. There is no mention in the result section of the abstract regarding the lack of resources in Ethiopia. There is also nothing in the result section of the abstract that discusses the care preterm babies receive at home or in facilities. I will also be cautious about using the term misconceptions for the following reasons. A) The approved age of viability in Ethiopia may be different from the US, so a 27-week gestation fetus is technically a pregnancy loss, not the death of a baby. It is unclear from the abstract what preterm gestation was examined.

Main manuscript:

Introduction:

Can the authors provide more updated statistics than those from 2019? (https://www.who.int/news-room/fact-sheets/detail/newborn-mortality)

I think you would need a reference for the 90% statistic you quote for the three causes of preterm deaths in Ethiopia.

The last statement in paragraph 2 of the introduction (line 92) is a repeat of the second statement with numbers (like 89).

In general, the introduction does more to describe the burden and complications of preterm birth in SSA than it does to lay the foundation for the study. Only in the last paragraph do we have justification for the study. Even then, it is not well described/justified (in my opinion).

Methods:

Design: Might this be a cross-section? Also, including the justification of why you chose this study design, and lastly, the guiding theory that supports your study.

Setting: Please consider replacing 'site' with 'community'.

Study participants: It is not clear if door-to-door (in the community) recruitment occurred, but it is stated that home interviews occurred. It is also not clear if recruitment occurred at

facilities or if the potential participants were identified from the hospital registry and then approached at home or in a different setting for recruitment into the study. Please provide more clarity on where and how each study group was recruited. For example, did the family of the index mother with a preterm birth include only the people at home at the time of the interview, or were they recruited differently/interviewed differently from the index mother of a preterm infant? It would also be good to provide some definition of who/what a community stakeholder is/implies.

Data management: (line 185). Can the authors please provide the specific research question that guided the coding process? There could also be some more elaboration on the coding steps. For example, initial codes were developed and reviewed before being applied to other transcripts.

My interpretation is that the individual perception will involve participants who have had a preterm infant, the interpersonal level will include results from immediate family, the organization will involve health workers, and the societal level will involve stakeholders. Is that correct? If, yes, that is not clearly communicated in the methods section.

Given that countries typically have age of viability, did you exclude discussing with parents with births that were below the age of viability, which would technically be an abortion and not a birth (per the laws of the land).

Was there any restriction by age, because I see the age of participants included teenagers?

Results:

I would suggest the authors present their results as major themes that fall under the different socioecological groups.

As commented on in the methods, the Individual level of SEM refers to the individual's perception, interpersonal involves interactions with other proximal to the individual, etc. However, the way the results are presented, under the individual, there are themes/quotes from sister in-laws and grandmothers who technically do not fit in the individual level of the SEM model.

Discussion: The discussion does not engage with the literature well enough and for the most part it is repeating the results rather that providing potential rational for the findings.

I also think that there is nothing in the results section that directly links perception to care provision. Without describing the clinical capacity of the hospitals in the community this study was conducted in, it would be misleading to statements such as : “We argue that these perceptions, beliefs, and attitudes towards the mother-infant dyad, particularly in cases of preterm birth, cut across individual, interpersonal, organizational, and societal levels. They in turn impact home healthcare practices, the healthcare-seeking behavior of families, the ability to use community support systems, the availability of healthcare services at facilities, and the motivation and will of healthcare providers to deliver these services to preterm mother-infant dyads”.

There are also several statements in the discussion that were not derived from the results of the study: Example: “This bias is driven by the providers’ desire to shield parents from the burden of caring for a child who may survive with disabilities, and to prevent the infant from experiencing suffering” If this is a statement from a prior publication then a reference should be provided.

There is also no discussion of study limitations.

Conclusion: Yes, there are evidence-based interventions to improve the survival of preterm infants. However, most of the gains regarding these interventions are not yet realized in LMICs such as Ethiopia, especially (if I am correct), rural regions where resources to care for preterm infants would be more lacking. The conclusions drawn from the results do not seem to be reflective of the results but rather the authors impression of what should be.

VERSION 1 - AUTHOR RESPONSE

Reviewer: 1 Dr. Thais Silva, State University of Campinas, Centro Integrado de Saúde Amaury de Medeiros	
Comments to the Author:	
This is a very interesting analysis, since most part of prematurity worldwide comes from low-income countries, which makes urgent to understand possible cultural and educational barriers not only for parents, but also for the community and caregivers. Without such specific characteristics related to social relations, it is almost impractical to introduce preventable actions in different populations waiting for the same reductions in prematurity rate. Therefore, this qualitative analysis can be helpful to clarify some aspects to guide future target interventions for this population. However, I would like to have some clarifications in order:	We appreciate the reviewer’s positive feedback on the relevance of the paper.
1) How was defined the total sample? Which criteria were used to select participants using the hospital registers? The investigator included all preterm birth registers? How was the recruitment for focus group discussions?	Thank you for this important comment. We purposively selected participants to gain rich information on experiences of mothers of preterm newborns, their family and care providers and stakeholders influencing the care provision. We added text describing our initial planned sample size and a table displaying the numbers per

	participant group (Table 1). Lines 182 – 183
2) The semi-structured guides used to conduct the in-depth interviews and the focus group discussions should be available on supplementary information. I would like to evaluate the questions, points and addressed issues contained on these guides.	Thank you for your comment. We have now attached the IDI and FGD guides as supplementary materials.
3) Table 1: Line 54 Wouldn't be better also use the term "women" despite "mother" as done in Line 51? The manuscript do not include how many interviewed women experienced the loss of their child. Would be interesting to have this information.	Thank you for your feedback. We agree with the suggestion; we replaced the word 'mothers' with 'women'. We didn't include mothers whose neonates were diseased due to ethical issue.
4) Line 374: It was difficult to understand if the preterm delivery rate on health centers is small or if the health care providers do not understand this rate clearly. It should be clearer. If the preterm delivery rate is really small, why do not recruit professionals from health centers with a higher number of preterm births on that region?	Thank you for your comment. In general, health centers tend to have a low delivery caseload due to the relatively small catchment population they serve - about 25,000 people on average. However, they collectively account for a significant share of facility-based births in Ethiopia, contributing between 60–70%. This is largely because of their sheer number (approximately 4,000 centers compared to around 400 hospitals) and their accessibility to the majority of pregnant women, who predominantly live in rural areas.<br><br>To capture the perceptions and beliefs of providers across different levels of the health system, we included participants from both health centers and health posts, the latter being one tier below health centers.
5) The discussion section does not include strengths and limitations. Please, include this information.	Thank you for your comment. We have now included 'strengths and limitations' in the revised version, in place of 'key messages' as suggested by the editor. Lines 63-77
Reviewer: 2<br>Dr. Osayame Ekhaguere, Indiana University Department of Pediatrics	

Comments to the Author:	
Thank you for the opportunity to review this manuscript. The topic is very relevant to understanding parental and societal perceptions of preterm births. The justification for conducting the study are strong. The methods could be improved by providing information on current policies defining “preterm vs abortion" in Ethiopia and describing the clinical capacity of the health facilities that cater to the study community. Only by understanding the context in which the study was conducted can the results be interpreted and understood completely. While the results using the SEM is good, the results would be been more organized if theme under each branch of the SEM were provided. Finally, are most important, the conclusions of the study do not necessarily reflect the findings of the study. Specifically, there is nothing in the results that suggests home healthcare practices, the healthcare-seeking behavior of families are influenced if the baby is premature or that healthcare services at facilities, and the motivation and will of healthcare providers to deliver these services to preterm are impeded by their perception of preterm birth. They may be influenced by the availability to provide these services, but this was not explored in the study so the conclusion cannot be made. See below specific comments.	Thank you so much for the detailed and insightful review of our manuscript. Your review gave us the opportunity to strengthen the quality of our paper.
Below are my comments regarding the different sections of the write-up.	
Title: I think the title is over sensationalized, especially given the statement was not universally echoed by the study participants. One quote from a stake holder (not even an individual who has experienced the personal loss of a preterm infant) should not dictate the title of a publication.	Thank you for your suggestion. We agree with the suggestion; we revised the title by dropping the quote.
Abstract:	
Methods: The way Batu and Meki are described as sites makes it look like they are institutions in which the study was	Thank you for pointing this out. We agree with the suggestion; we updated the wording accordingly. Linn 36

conducted. Please consider replacing site with community.	
Results: It seems there is a typographical error in the first statement of the result section. "Giving {birth seems to be missing} to a preterm birth is often associated with fear, stress, unhappiness, concern, and worry".	Thank you for your suggestion. We updated the wording accordingly. Line 44
I would have expected the results section to have a major theme for each level of the ecological model.	Thank you for the suggestion. Because of the word limit we found it difficult to present major theme for each SEM component in the abstract.
Conclusion: The conclusion should reflect the results that are shown. There is no mention in the result section of the abstract regarding the lack of resources in Ethiopia. There is also nothing in the result section of the abstract that discusses the care preterm babies receive at home or in facilities. I will also be cautious about using the term misconceptions for the following reasons. A) The approved age of viability in Ethiopia may be different from the US, so a 27-week gestation fetus is technically a pregnancy loss, not the death of a baby. It is unclear from the abstract what preterm gestation was examined.	Thank you for these insightful comments. You are right, lack of resources was not presented in the findings of the abstract and was not the primary objective of the study either. We removed it from the conclusion. Lines 56-59
Main manuscript:	
Introduction:	
Can the authors provide more updated statistics than those from 2019? (https://www.who.int/news-room/fact-sheets/detail/newborn-mortality)	Thank you for the suggestion. We agree with the comment, and we have now updated the figures with more recent data. Lines 80-85
I think you would need a reference for the 90% statistic you quote for the three causes of preterm deaths in Ethiopia.	Thank you for the comment. We added citation for the figure. Line 91
The last statement in paragraph 2 of the introduction (line 92) is a repeat of the second statement with numbers (like 89).	Thank you for the comment. We clarified the last statement – it refers to the cause of death among preterm neonates while the second statement refers to the cause of death among all newborns. Lines 92-93
In general, the introduction does more to describe the burden and complications of preterm birth in SSA than it does to lay the foundation for the study. Only in the last paragraph do we have justification for the	Thank you for your insightful comment. We revised the introduction section adding text that lay the foundation for the study.

study. Even then, it is not well described/justified (in my opinion).	
Methods:	
Design: Might this be a cross-section? Also, including the justification of why you chose this study design, and lastly, the guiding theory that supports your study.	We appreciate this insightful comment. We have updated the study design based on this suggestion. Lines 145-153
Setting: Please consider replacing 'site' with 'community'.	Thank you again, we replaced site with community. Line 156
Study participants: It is not clear if door-to-door (in the community) recruitment occurred, but it is stated that home interviews occurred. It is also not clear if recruitment occurred at facilities or if the potential participants were identified from the hospital registry and then approached at home or in a different setting for recruitment into the study. Please provide more clarity on where and how each study group was recruited. For example, did the family of the index mother with a preterm birth include only the people at home at the time of the interview, or were they recruited differently/interviewed differently from the index mother of a preterm infant? It would also be good to provide some definition of who/what a community stakeholder is/implies.	Thank you again for the very useful comment. We revised the participants' description, added table 1 summarizing the IDI and FGD sample size planned, and updated the text describing the places where interviews were conducted. Lines 166 - 196
Data management: (line 185). Can the authors please provide the specific research question that guided the coding process? There could also be some more elaboration on the coding steps. For example, initial codes were developed and reviewed before being applied to other transcripts.	Thank you for this comment. We added the research question that guided the coding process. Lines 225-228
My interpretation is that the individual perception will involve participants who have had a preterm infant, the interpersonal level will include results from immediate family, the organization will involve health workers, and the societal level will involve stakeholders. Is that correct? If, yes, that is not clearly communicated in the methods section.	Thank you for pointing this out. We agree with you, and we added a text linking the study participants' groups with the SEM components. Lines 233-240

Given that countries typically have age of viability, did you exclude discussing with parents with births that were below the age of viability, which would technically be an abortion and not a birth (per the laws of the land).	Thank you for highlighting this. The gestational age of viability in Ethiopia is not consistently defined both in guidelines and practices. Maternal healthcare guidelines and providers often apply 28 weeks of gestation as age of viability. Neonatal healthcare guidelines and providers apply 26 weeks as age of viability and do still recommend consideration of resuscitation for 25 week in some circumstances. We added this text in the introduction (Lines 113-119) and reflected on its implication in the discussion section.
Was there any restriction by age, because I see the age of participants included teenagers?	Thank you for your feedback. There was no age restriction for mothers. Ethiopia's national ethics review guideline states that "Emancipated minors – working or earning their living, married, parenting – may be allowed to give an informed consent or an IRB may decide a waiver of consent".
Results:	
I would suggest the authors present their results as major themes that fall under the different socioecological groups.	Thank you for your helpful suggestion. We thematized the results under the SEM in the revised version.
As commented on in the methods, the Individual level of SEM refers to the individual's perception, interpersonal involves interactions with other proximal to the individual, etc. However, the way the results are presented, under the individual, there are themes/quotes from sister in-laws and grandmothers who technically do not fit in the individual level of the SEM model.	Thank you for pointing this out. In the revised version, we moved the themes/quotes from family members that were previously presented under the individual level. However, at the interpersonal, organizational, and societal levels, we still retained some themes/quotes from study participants that fall 'outside these SEM components,' as it is difficult to exclusively position participants and their perspectives within a single SEM category.
Discussion: The discussion does not engage with the literature well enough and for the most part it is repeating the results rather that providing potential rational for the findings.	Thank you for this comment. We agree and we reviewed the discussion connecting our study findings with what was documented in previous literature and reflect on the implications.

I also think that there is nothing in the results section that directly links perception to care provision. Without describing the clinical capacity of the hospitals in the community this study was conducted in, it would be misleading to statements such as : “We argue that these perceptions, beliefs, and attitudes towards the mother-infant dyad, particularly in cases of preterm birth, cut across individual, interpersonal, organizational, and societal levels. They in turn impact home healthcare practices, the healthcare-seeking behavior of families, the ability to use community support systems, the availability of healthcare services at facilities, and the motivation and will of healthcare providers to deliver these services to preterm mother-infant dyads”.	Thank you for this insightful feedback. We agree, the results and discussion section didn’t explicitly link beliefs, perceptions, and attitudes surrounding preterm birth with care provision. We revised the results and discussion section to make the message more explicit. We added text on the health system’s limitation for provision of quality care for preterm newborns.
There are also several statements in the discussion that were not derived from the results of the study: Example: “This bias is driven by the providers’ desire to shield parents from the burden of caring for a child who may survive with disabilities, and to prevent the infant from experiencing suffering” If this is a statement from a prior publication then a reference should be provided.	Thank you for these comments. We have reviewed the discussion to clarify what comes from our data and included citations for those drawn from literature
There is also no discussion of study limitations.	Thank you for your comment. We have now included ‘strengths and limitations’ in the revised version, in place of ‘key messages’ as suggested by the editor. Lines 63-77
Conclusion: Yes, there are evidence-based interventions to improve the survival of preterm infants. However, most of the gains regarding these interventions are not yet realized in LMICs such as Ethiopia, especially (if I am correct), rural regions where resources to care for preterm infants would be more lacking. The conclusions drawn from the results do not seem to be reflective of the results but rather the authors impression of what should be.	Thank you for your comment. We revised the conclusion to make it consistent with the discussion. Lines 731-738
